# *Pleurotus djamor* Mycelium: Sustainable Production of a Promising Protein Source from Carrot Side Streams

**DOI:** 10.1021/acs.jafc.5c11223

**Published:** 2025-12-17

**Authors:** Leonie Cora Juhrich, Iris Lammersdorf, Pascal Schmitt, Lars Tasto, Falk Speer, Denise Salzig, Kai Reineke, Holger Zorn, Martin Gand

**Affiliations:** † Institute of Food Chemistry and Food Biotechnology, 9175 Justus Liebig University Giessen, Heinrich-Buff-Ring 17, Giessen 35392, Germany; ‡ Institute of Bioprocess Engineering and Pharmaceutical Technology, University of Applied Sciences Middle Hesse, Gutfleischstrasse 3-5, Giessen 35390, Germany; § 365994 GNT Europa GmbH, Kackertstrasse 22, Aachen 52072, Germany; ∥ Fraunhofer Institute for Molecular Biology and Applied Ecology, Ohlebergsweg 12, Giessen 35392, Germany

**Keywords:** screening, basidiomycota, fermentation, mycelium, upcycling, novel foods, *Pleurotus djamor*, side stream, meat analog

## Abstract

Innovative protein sources are urgently needed to feed a growing global population and to support the increasing shift toward vegetarian and vegan lifestyles. Mycelia of edible fungi offer a sustainable and efficient alternative food source. In this study, 106 fungal strains were explored for their ability to ferment two different liquid carrot side streams. Among the candidates, *Pleurotus djamor* demonstrated exceptional potential, with high yields of biomass of ∼15 g L^–1^ and high protein contents of 31.0 ± 5.9 (optimized orange carrot medium) or 21.6 ± 1.9 g 100 g^–1^ (optimized black carrot medium), respectively. When used in burger patties and vegan sausage analogs, the mycelia outperformed vegetable proteins in sensory tests, highlighting their viability as a nutritious, versatile, and consumer-accepted protein alternative.

## Graphical abstract

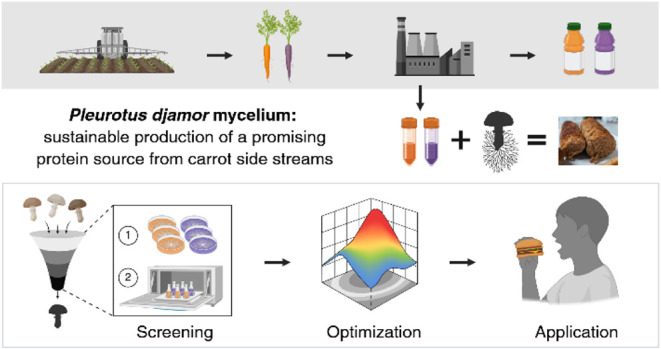

## 1 Introduction

Ensuring a sustainable nourishment of a global population projected to reach 9–10 billion by 2050, while minimizing environmental impacts such as greenhouse gas emissions, biodiversity loss, land degradation, and ecosystem disruption, stands as one of the most pressing challenges of the 21st century.^1^ In 2019, about 9% of the world’s population suffered from starvation, and 29% were affected by food insecurity. These issues have worsened in the last years due to the growth of the human population, exacerbated by the outbreak of multiple wars and resulting humanitarian crises regions such as Ukraine, Gaza, and Sudan.^2^

To address the challenges of feeding a growing global population, there is a need to increase food productivity. An alluring option involves implementing alternative sustainable production methods for protein sources. This approach aligns with the global shift toward vegetarian and vegan lifestyles, as the renunciation of traditional animal-based products gains momentum.^3^ While plant-based proteins are well-established alternatives, increasing attention has been directed toward fungal mycelia, the vegetative hyphal network of edible mushrooms, as a new food source over the last years. Mycelia offer significant advantages over mushroom fruiting bodies, including shorter cultivation periods and reduced spatial requirements.^4,5^ The mycelia often have only minimal flavor, are nearly colorless, and show good chewing properties as well as a meat-like structure.^6–8^ Filamentous fungi, as natural decomposers, have the remarkable ability to break down almost every organic material, including indigestible fibers such as cellulose and hemicellulose. This capability allows for utilizing side streams generated by the current food production systems, providing a sustainable solution for cultivating mycelia while upcycling materials that would otherwise be wasted. Importantly, these side streams can still maintain food-grade quality, making them viable for further applications. Examples include whey, apple pomace, and vinasse, which have been successfully explored for the cultivation of fungi.^9,10^ Several factors influence fungal growth, which can broadly be categorized as physical, chemical, and biological. This study focused on chemical factors, particularly the pH value and the carbohydrate content (and overall nutrient composition) of the culture medium, using two liquid carrot side streams, which come from the production of natural colors from black and orange carrots.

Side streams, if left unutilized, generate waste, leading to environmental concerns, as well as increased costs for their disposal. However, when they are managed appropriately, side streams can be transformed into valuable food and feed resources. While solid substrates, such as pomace, are especially suitable for surface cultivation, liquid side streams may be used for submerged cultivation, leading to protein-rich fungal mycelia.^11–13^

A comprehensive screening process was conducted, growing 106 fungal strains in surface cultures, and subsequent screening of 22 well-growing strains in submerged cultures. Unlike traditional optimization methods, such as the one-factor-at-a-time approach, which often overlooks interactions between parameters, Response Surface Methodology (RSM) was used in this study to optimize to culture conditions. RSM enabled efficient optimization while minimizing the number of experiments, ensuring a more robust and systematic approach to improve the outcome.^14,15^ Compared with the screening conditions, an optimization of all parameters was achieved for nearly all fungus-medium combinations. Especially, the dry matter increased by a factor of 2.6 for *Pleurotus djamor* (PDJ) grown on orange carrot medium, while the crude protein content increased by a factor of 1.3 on the black carrot-based medium. The mycelia obtained after optimization of the culture conditions were incorporated in vegan burger patties and sausage-like food to demonstrate their applicability in food products. The mycelium-based patties gained a higher overall likeliness than patties containing soy protein isolates, highlighting mycelia as a promising alternative to plant-based proteins with comparable nutritional parameters.

## 2 Material and Methods

### 2.1 Chemicals

The following chemicals were obtained from Carl Roth GmbH + Co KG (Karlsruhe, Germany): *N*-acetylglucosamine (>99%), agar–agar Kobe I (for microbiology), *p*-aminohippuric acid (>98% for biochemistry), bromothymol blue (sodium salt p.a.), chitin (from crab shells), citric acid (>99.5%), copper sulfate pentahydrate (>98%, cryst.), fructose (>99.5% for biochemistry), galactose (>98% for biochemistry), glucose monohydrate (for microbiology), iron­(III) chloride (free of water, purest, >98.5%), maltose monohydrate (>95% for biochemistry), phosphoric acid (85%), sodium chloride (>99.8%), sodium hydroxide (>98%), sucrose (p.a. >99.5%), urea (for analysis, >99.5%), and zinc acetate dihydrate (>99%). Acetic acid, catalyst CT 5.3 g, glacial acid, and sodium thiosulfate solution (0.1 M) were purchased from VWR International LLC (Leuven, Belgium). Ammonium sulfamate (>98%), petroleum ether (deep boiling), and phthalic acid (>99.5%) were supplied by Fisher Scientific GmbH (Schwerte, Germany). Th. Geyer GmbH & Co KG (Renningen, Germany) supplied boric acid (>99.8%), dichloromethane (>99.5% for HPLC), formic acid, hydrochloric acid (0.1 M; purest, 25%; for analysis, 37%), hydrogen peroxide (30%), malt extract, sodium carbonate, sulfuric acid (for analysis, 98%), and trisodium citrate dihydrate. Ethanol (technical) was purchased from Stockmeier Holding SE (Bielefeld, Germany). The β-glucan enzyme assay kit (for yeast and mushroom, K-YBGL) was procured from Megazyme Ltd. (Wicklow, Ireland). Ethylenediaminetetraacetate (>99%), lactose monohydrate, and sulfuric acid (for analysis, 72%) were supplied by AppliChem GmbH (Darmstadt, Germany). Merck KGaA (Darmstadt, Germany) provided peptone (from soy), phenol, potassium iodide (pure), sodium nitrite (for analysis), and trichloroacetic acid (for analysis). Sodium metabisulfite was obtained from Bernd Kraft (Duisburg, Germany), and sodium hydroxide solution (32–33%) was sourced from Otto E. Kobe KG (Marburg, Germany). Potassium hexacyanidoferrate (II) (>98%) was purchased from Alfa Aesar GmbH & Co. KG (Karlsruhe, Germany). β-Mercaptoethanol and thiodiglycol (>95.0%) were acquired from Sigma-Aldrich Chemie GmbH (Taufkirchen, Germany), and 3-methyl-2-benzothiazolinon-hydrazonhydrochlorid was obtained from Fluorochem Ltd. (Hadfield, United Kingdom). Sunflower oil (refined) was obtained from Bröckelmann & Co. (Oelmühle, Hamm, Germany). Soy granulate (texturized) was purchased from Velivery GmbH & Co. KG (Nabburg, Germany), and soy protein isolate (92%) was sourced from Piowald GmbH (Mühbrook, Germany). Food colorants, including Fiesta Pink and Brilliant Orange, were obtained from GNT Europa GmbH. Binder (vegan binder 304162) was obtained from Food Ingredients & Specialties (Maastricht, Netherlands). Liquid black carrot (pH 3.6) and orange carrot (pH 4.2) filtration side streams were provided from GNT Group B.V. (Mierlo, Netherlands). Ninhydrin, sodium citrate buffer, 0.12 N, pH 3.4, and sodium citrate buffer, 0.20 N, pH 10.8, were purchased from Sykam Chromatographie Vertriebs GmbH (Fürstenfeldbruck, Germany). Gluten (100% wheat gluten) was bought from Sharkfood Nutrition (Vogtsburg, Germany). Chickpeas (King’s Crown) and garlic powder (Le Gusto) were purchased from Aldi Süd (Giessen, Germany). Tomato paste (MUTTI, 2-times concentrated) was bought at Rewe (Giessen, Germany). Table salt (Safrisalz, gritty) was obtained from Kaufland GmbH (Giessen, Germany). Pepper (black, whole) and paprika powder (sweet) were bought at Turgut Markt (Marburg, Germany).

### 2.2 Microorganisms

106 fungal strains were screened on both side streams (see Table S1, Supporting Information). The Basidiomycota used in this work were supplied by the German Collection of Microorganisms and Cell Cultures GmbH (DSMZ, Braunschweig, Germany), the Westerdijk Fungal Biodiversity Institute (CBS, Baarn, Netherlands), the companies Mycelia NV (Deinze, Belgium), Sylvan (Langeais, France), InterMed Discovery (Dortmund, Germany), and Steintaler Edelpilze (Neu Wulmstorf, Germany), Georg August University of Göttingen (Göttingen, Germany), Kyoto University (Yoichi Honda, Kyoto, Japan), Fraunhofer-Institute for Molecular Biology and Applied Ecology IME (Giessen, Germany), and the culture collection of the Justus Liebig University Giessen (LCB, Giessen, Germany). For strain maintenance, agar stocks of all fungi were kept on either malt extract agar (malt extract 20 g L^–1^, agar–agar 15 g L^–1^) or 20% malt extract peptone agar plates (malt extract 20 g L^–1^, peptone 3 g L^–1^, agar–agar 15 g L^–1^) at 24 °C in the dark (see Table S1, Supporting Information).

### 2.3 Culture Conditions

#### 2.3.1 Surface Screening

The side streams (GNT Group B.V., Mierlo, Netherlands) (for composition, see Table S2, Supporting Information) were diluted to a 2.2% carbohydrate (CH) (orange carrot) and 1.8% CH (black carrot) content. Due to their low initial pH values, the side streams were autoclaved separately from the agar–agar (30 g L^–1^) and mixed afterward. The Petri dishes had a diameter of 94 mm and a height of 16 mm (Greiner Bio-One, Kremsmünster, Austria). For cultivation, an overgrown agar piece with a diameter of 0.8 cm from the strain-maintenance plate was transferred to the orange carrot agar (OCA) or black carrot agar (BCA) plate, respectively. The growth of the mycelium was monitored in regular intervals over 14 days in duplicates, and the sensory impression was evaluated by a panel of five untrained panelists.

#### 2.3.2 Screening in Submerged Cultures

For the precultures, a 0.2 cm^2^ with mycelium overgrown piece of an agar plate was transferred to an Erlenmeyer flask (250 mL) containing 100 mL of autoclaved malt extract peptone or malt extract medium and homogenized by an Ultra-Turrax T25 homogenizer (IKA, Staufen, Germany) for 30 s at 10 000 rpm. The cultures were incubated at 150 rpm in darkness at 24 °C for 7 days. For the main cultures, the same volume and flasks were used, while the side streams were diluted to 2.2% CH for the liquid orange carrot medium (OCM) and 1.8% CH for the black carrot medium (BCM), both with their natural pH and inoculated with 10% (v/v) homogenized precultures. The cultivation was performed in the dark at 24 °C and 150 rpm for 10 days.

Twenty-two and 17 strains were screened in submerged cultures in OCM or BCM, respectively (Table S3, Supporting Information). The screening lasted for 10 days, and the mycelia were harvested in 50 mL Falcon tubes (Fisher Scientific GmbH) each day by centrifugation with a Megafuge 16R (Fisher Scientific GmbH) at 4200 × *g* at room temperature for 10 min. The supernatant was decanted, and the process was repeated, until no supernatant was left. Dry matter and sensory attributes were investigated each day. Furthermore, on the day with the highest dry matter, the crude protein content was determined.

### 2.4 Determination of Dry Matter

To determine the dry matter (DM) during the screening in submerged cultures, the wet mycelia were weighed and the moisture content was determined using a moisture analyzer (Kern & Sohn GmbH, Balingen-Frommern, Germany). The dry matter was calculated using eq 1.

1 $$DM_{\mathrm{mycelia}}=\left(\left(m_{\mathrm{full}}-m_{\mathrm{empty}}\right)\times \left(100-M\right)\right)\times 10$$

*M*: moisture [%]

*m*_full_: Falcon tube with mycelia [g]

*m*_empty_: empty Falcon tube [g]

DM_mycelia_: dry matter [g L^–1^]

10: conversion factor in L

To determine the dry matter during the optimization, the mycelia were lyophilized for approximately 4 days using an Alpha 1–2 LD Plus (Martin Christ Gefriertrocknungsanlagen GmbH, Osterode am Harz, Germany) lyophilizer and weighed. The dry matter was calculated using eq 2.

2 $$DM_{\mathrm{mycelia}}=\frac{m_{\mathrm{full}}-m_{\mathrm{empty}}}{V}$$

*m*_full_: Falcon with mycelia [g]

*m*_empty_: empty Falcon tube [g]

*V*:volume of side stream [L]

DM_mycelia_: dry matter [g L^–1^]

### 2.5 Crude Protein Content Determination

To determine the crude protein (CP) contents of lyophilized mycelia, the Kjeldahl method was used. The Kjeldahl factors were 4.50 for screening and optimization, 5.93 for PDJ on the optimized orange carrot medium (OCO), and 5.80 for PDJ on the optimized black carrot medium (BCO) after determination through amino acid analysis. A detailed description can be found in the Supporting Information.

### 2.6 Design of Experiment

After identifying 10 well-growing fungus-medium combinations, an optimization of the culture conditions was performed by varying the pH and the CH content to increase the yields of DM and CP contents. For the Design of Experiment (DoE), the software Design-Expert (StatEase, Inc.) with a central composite design (CCD) was used. For randomized quadratic models, D-optimal designs with six center points (CP), three factorial points, two star points, and eight lack-of-fit (LoF) points were generated. The limits of the side streams for DoE were tested beforehand (data not shown), resulting in the following ranges: The CH content ranged from 0.6% to 6.7% for the orange carrot substrate and from 0.4% to 5.4% for the black carrot substrate (Table S4, Supporting Information). The pH ranged between 3 and 9, while more extreme values can cause lysis of fungal cells. This led to 34 runs for each fungus-medium combination. An extension of the DoE for PDJ in BCM (CH up to 7.2% and pH up to 12–0) and *Pleurotus geesterani* (PGE) in OCM (CH content up to 11.2%) was made.

The precultures were cultivated in 1 L flasks as described above. For the main cultures, the substrate was prediluted to 10% CH content and autoclaved. The pH was adjusted using 0.5, 1, and 6 M NaOH as well as HCl, and the media were diluted with sterile water to the final concentration, followed by sterile filtration with X12 Bottle-Top filters 500 mL polyether sulfone (PES) with a pore size of 0.2 μm (Fisher Scientific GmbH) and a pressure of 150 mbar. For the main cultures, 10% (v/v) homogenized preculture was used as inoculum. The cultivation time was specific for each fungus-medium composition (Table S3). The cultivation was performed in the dark at 24 °C and 150 rpm. The mycelia were harvested, and the CP was determined as described above. The response surfaces were calculated, and the media yielding the highest dry matter and CP contents were predicted. To validate these parameters, three replicates were performed using the same cultivation parameters as those used before. Furthermore, two controls using the parameters of the medium during the screening were run in parallel.

### 2.7 Nutritional Composition Analysis

*P. djamor* was cultivated under optimized conditions in 2 L flasks. Optimized black carrot medium (BCO): 4.2% CH and a pH of 6.6 as well as optimized orange carrot medium (OCO): 6.0% CH and a pH of 4.8. DM and CP contents were measured as described above. The ash content was determined gravimetrically. The fat content was determined by the method of Weibull-Stoldt and the chitin content was determined as described by Ahlborn et al.^11^ The glucan contents were measured using a β-glucan assay kit for yeasts and mushrooms. The amino acid analysis was performed as described by Ahlborn et al. using 25 mg of lyophilized mycelium.^11^ The contents of reducing sugars and sucrose were determined using the method of Luff-Schoorl. Detailed descriptions are provided in the Supporting Information.

### 2.8 Sensory Evaluation of the Mycelia

The sensory attributes were evaluated by an untrained panel of 15 panelists in one session with duplicates. The panel members were between 22 and 35 years old, with 5 male and 10 female panelists. The panel described the optical properties as well as the texture of the fresh mycelia. Furthermore, smell and taste were rated from 0 (neutral) to 5 (high intensity). For the olfactory evaluation, the following attributes were used: fruity, carrot, sweet, sour, fungal, smoky, and earthy, while carrot, sweet, sour, fungal, bitter, meat-like, salty, and spicy were chosen as gustatory attributes (data not shown). The authors confirm that all sensory evaluations were conducted in strict adherence to the ethical principles outlined in the World Medical Association’s Declaration of Helsinki for research involving human participants. In the context of sensory evaluation, national regulations do not mandate formal ethical approval, and there is no established ethics committee or formal documentation process required for such studies. Nevertheless, the authors implemented stringent protocols to uphold the privacy of all of the participants. These protocols included ensuring voluntary participation without any coercion, providing detailed information about the study’s objectives, procedures, and potential risks, obtaining verbal informed consent from each participant, ensuring that no participant data were disclosed without prior consent, and allowing participants the freedom to withdraw from the study at any time without any adverse consequences.

### 2.9 Food Application

For the patties, 40 g of soy granulate was soaked in 116 mL of water (with natural colorants from GNT Group B.V.) and incubated until all water was absorbed. In total, 14 g of binder, 8 g of soy protein isolate, 4 g of glucose monohydrate, and 3 g of sodium chloride were mixed with 15 g of sunflower oil. The soy protein isolate was substituted with 25%, 50%, 75%, and 100% of the mycelium. Afterward, the swollen soy granulate was added, and the mixture was mixed. The patties were fried at medium heat until they were brown for 3–5 min in sunflower oil.

The patties were evaluated by 15 untrained panelists (22–37 years old, nine females, six males). The panel described the visual appearance and texture of the patties. Smell, taste, and overall impression were rated on a scale from 0 to 5. For gustatory evaluation, the categories were sweet, sour, fungal, bitter, salty, and meat-like. For olfactory evaluation, the categories were carrot, sweet, sour, fungal, and smoky.

For the vegan sausages, 100 g of soaked chickpeas or fresh mycelium, 2 teaspoons of tomato paste, salt, pepper, garlic powder, and paprika powder were blended at level 10 in a Thermomix TM6 (Vorwerk SE & Co. KG, Wuppertal, Germany). Afterward, 100 g of gluten was added to the paste inside the Thermomix and mixed at level 1. The resulting paste was filled in cloth strainer, formed into sausages, and cooked in boiling water for 30 min. After cooling, the sausages were fried in 3 tablespoons of sunflower oil for 7 min until golden brown.

The sausages were evaluated by 18 untrained panelists (22–47 years old, nine females, eight males). Smell and taste were rated from 0 to 5. For gustatory evaluation, the selected attributes were carrot, nutty, sweet, fungal, bitter, umami, salty, meat-like, and vegetable. Furthermore, the panel could name additional attributes. For the olfactory evaluation, the categories were carrot, nutty, sweet, fungal, smoky, earthy, and vegetable. Furthermore, the panel could name additional attributes. Only descriptive tests were used.

### 2.10 Statistical Analyses

For the significance test in food applications, a paired two-sample *t* test was performed using the XLMiner Analysis ToolPak in Microsoft Excel.

## 3 Results and Discussion

### 3.1 Surface Screening

In the surface screening of 106 fungi, only one strain (*Clitocybe odora*) failed to grow on OCA, while 18 fungi did not grow on BCA (growth of 0.8 ± 0.0 cm) (Table 1). On the 10th culture day, 32 fungi fully overgrew the OCA and eight the BCA plates, respectively. Based on the results of the surface culture screening, 17 fast-growing (growth of over 4.2 cm on day 10 of cultivation) fungi with neutral to savory sensory olfactory characteristics were selected for further submerged culture screening in BCM and 22 in OCM, respectively.

**Table 1:** Growth on the 10th Day of Surface Cultivation of All Strains on Orange Carrot Agar (OCA) and Black Carrot Agar (BCA)^a^

Fungus	Growth [cm] on OCA	Growth [cm] on BCA	Fungus	Growth [cm] on OCA	Growth [cm] on BCA
*Abortiporus biennis*	4.5 ± 0.1	3.4 ± 0.6	*Meripilus giganteus I*	7.6 ± 0.5	4.9 ± 0.4
*Agaricus arvensis*	4.2 ± 0.1	1.0 ± 0.3	*Meripilus giganteus II*	8.5 ± 0.0	5.6 ± 0.2
*Agaricus bitorquis*	2.2 ± 0.2	0.8 ± 0.0	*Merulius tremellosus*	8.5 ± 0.0	8.5 ± 0.0
*Amylostereum chailletii*	1.0 ± 0.3	1.0 ± 0.1	*Mycena pseudocorticola*	4.5 ± 0.1	3.7 ± 0.2
*Armillaria bulbosa*	4.7 ± 1.7	1.3 ± 0.5	*Mycetinis scorodonius*	7.4 ± 0.4	5.3 ± 0.2
*Armillaria gallica*	2.2 ± 0.3	1.4 ± 0.1	*Neolentinus lepideus*	6.8 ± 0.3	3.3 ± 0.0
*Armillaria mellea*	2.4 ± 0.4	1.4 ± 0.1	*Phanerochaete chrysosporium*	4.6 ± 0.3	3.8 ± 0.2
*Armillaria tabescens*	3.7 ± 1.0	2.6 ± 0.5	*Phlebia centrifuga*	8.5 ± 0.0	8.5 ± 0.0
*Auricularia fuscosuccinea*	2.6 ± 0.2	0.8 ± 0.0	*Pholiota lignicola*	4.4 ± 0.4	2.8 ± 0.0
*Bovista plumbea*	1.3 ± 0.1	0.8 ± 0.0	*Pholiota nameko*	8.2 ± 0.3	4.5 ± 0.4
*Calocybe gambosa*	8.5 ± 0.0	7.4 ± 0.1	*Pleurotus citrinopileatus*	5.8 ± 0.8	2.1 ± 0.4
*Ceriporiopsis resinascens*	7.4 ± 0.3	7.1 ± 0.1	*Pleurotus cornucopiae*	7.3 ± 0.2	2.7 ± 0.1
*Clitocybe gibba*	1.3 ± 0.1	1.3 ± 0.3	*Pleurotus cornucopiae* var. *citrinopileatus*	7.6 ± 0.5	2.3 ± 0.4
*Clitocybe odora*	0.8 ± 0.0	0.8 ± 0.0	*Pleurotus cystidiosus*	3.6 ± 0.7	1.5 ± 0.2
*Clitopilus hobsonii*	5.8 ± 0.3	4.5 ± 0.0	*Pleurotus djamor*	8.5 ± 0.0	5.2 ± 0.4
*Coprinellus flocculosus*	4.8 ± 0.3	0.8 ± 0.0	*Pleurotus dryinus*	1.2 ± 0.2	0.8 ± 0.0
*Coprinus cinereus*	0.9 ± 0.1	0.8 ± 0.0	*Pleurotus eryngii*	8.5 ± 0.0	2.4 ± 0.1
*Coprinus comatus*	2.3 ± 0.2	0.8 ± 0.0	*Pleurotus euosmus*	8.5 ± 0.0	1.4 ± 0.4
*Coprinus erythrocephalus*	6.9 ± 0.4	0.8 ± 0.0	*Pleurotus geesterani*	8.5 ± 0.0	5.9 ± 0.3
*Coprinus sterquilinus*	1.8 ± 0.3	0.8 ± 0.0	*Pleurotus nebrodensis*	6.9 ± 1.1	1.0 ± 0.2
*Coprinus xanthothrix*	8.5 ± 0.0	1.0 ± 0.1	*Pleurotus ostreatus I*	8.5 ± 0.0	5.0 ± 1.2
*Cyathus helenae*	1.6 ± 0.3	0.8 ± 0.0	*Pleurotus ostreatus II*	8.5 ± 0.0	4.3 ± 0.7
*Cyclocybe aegerita*	7.8 ± 0.2	3.5 ± 0.2	*Pleurotus ostreatus III*	8.5 ± 0.0	5.5 ± 0.2
*Cystostereum murrayi*	3.1 ± 0.2	1.3 ± 0.1	*Pleurotus ostreatus IV*	8.5 ± 0.0	2.4 ± 0.4
*Exidia glandulosa*	2.9 ± 0.2	1.5 ± 0.1	*Pleurotus ostreatus var. V*	8.5 ± 0.0	5.4 ± 0.5
*Fistulina hepatica*	5.2 ± 0.5	2.6 ± 0.2	*Pleurotus ostreatus var. VI*	8.5 ± 0.0	5.0 ± 1.1
*Flammula alnicola*	2.5 ± 0.0	1.3 ± 0.1	*Pleurotus ostreatus VII*	8.1 ± 0.4	3.5 ± 0.0
*Flammulina velutipes I*	7.0 ± 0.7	0.8 ± 0.0	*Pleurotus populinus*	4.7 ± 0.7	4.6 ± 1.6
*Flammulina velutipes II*	8.4 ± 0.1	3.2 ± 0.4	*Pleurotus pulmonarius I*	8.5 ± 0.0	3.4 ± 0.6
*Flammulina velutipes III*	8.5 ± 0.0	2.2 ± 0.1	*Pleurotus pulmonarius II*	6.9 ± 1.6	7.3 ± 0.1
*Fomitopsis betulinus*	8.5 ± 0.0	8.5 ± 0.0	*Pleurotus pulmonarius II*	8.5 ± 0.0	2.7 ± 0.2
*Ganoderma lucidum*	8.5 ± 0.0	8.5 ± 0.0	*Pleurotus pulmonarius III*	8.5 ± 0.0	4.2 ± 0.8
*Gloeophyllum abietinum*	4.4 ± 0.3	2.5 ± 0.1	*Pleurotus sajor-caju*	8.5 ± 0.0	5.1 ± 0.7
*Gloeophyllum odoratum*	3.4 ± 0.2	2.8 ± 0.1	*Pleurotus salmoneo-stramineus*	8.5 ± 0.0	4.7 ± 1.0
*Gloeophyllum sepiarium*	7.4 ± 0.4	3.6 ± 0.4	*Pleurotus sapidus*	8.5 ± 0.0	4.8 ± 0.3
*Gloeophyllum trabeum*	6.0 ± 0.2	4.0 ± 0.1	*Pleurotus spodoleucus*	8.5 ± 0.0	3.9 ± 0.2
*Hericium cirrhatum*	2.7 ± 0.2	1.9 ± 0.1	*Pleurotus tuberregium*	8.2 ± 0.3	1.9 ± 0.7
*Hericium coralloides*	8.1 ± 0.4	3.6 ± 0.2	*Polyporus squamosus*	3.1 ± 0.3	0.8 ± 0.0
*Hericium erinaceus*	6.9 ± 0.9	4.6 ± 0.5	*Psathyrella candolleana*	6.2 ± 0.8	1.0 ± 0.0
*Hericium flagellum*	1.4 ± 0.1	1.5 ± 0.1	*Punctularia atropurpurascens*	8.3 ± 0.2	6.0 ± 0.4
*Hymenopellis radicata*	8.5 ± 0.0	4.5 ± 0.2	*Punctularia strigosozonata*	8.3 ± 0.2	7.2 ± 0.4
*Hypsizygus tessulatus*	5.2 ± 0.4	0.8 ± 0.0	*Pycnoporus cinnabarinus*	7.0 ± 0.6	4.2 ± 0.1
*Kuehneromyces mutabilis I*	4.4 ± 0.1	1.9 ± 0.0	*Pycnoporus coccineus*	8.5 ± 0.0	7.0 ± 0.1
*Kuehneromyces mutabilis II*	5.6 ± 0.1	2.9 ± 0.5	*Pycnoporus sanguineus*	8.3 ± 0.2	6.5 ± 0.3
*Laetiporus persicinus*	8.5 ± 0.0	8.5 ± 0.0	*Sparassis crispa*	3.7 ± 0.2	2.3 ± 0.1
*Laetiporus sulphureus*	8.5 ± 0.0	8.5 ± 0.0	*Strobilurus esculentus*	6.0 ± 0.1	3.2 ± 0.2
*Lentinula edodes*	8.5 ± 0.0	7.1 ± 0.3	*Stropharia caerulea*	1.5 ± 0.5	1.2 ± 0.5
*Lepista nuda*	4.2 ± 0.5	2.5 ± 0.5	*Stropharia rugosoannulata I*	3.9 ± 0.1	3.0 ± 0.3
*Lycoperdon pyriforme I*	1.6 ± 0.1	0.8 ± 0.0	*Stropharia rugosoannulata II*	5.0 ± 0.3	2.4 ± 0.1
*Lycoperdon pyriforme II*	1.8 ± 0.1	0.8 ± 0.0	*Suillus variegatus*	1.3 ± 0.1	0.8 ± 0.0
*Macrolepiota procera I*	3.5 ± 0.0	1.7 ± 0.1	*Trametes ochracea*	8.5 ± 0.0	8.5 ± 0.0
*Macrolepiota procera II*	3.6 ± 0.2	2.3 ± 0.8	*Volvariella bombycina*	2.5 ± 0.5	0.8 ± 0.0
*Marasmius alliaceus*	3.8 ± 0.2	5.6 ± 0.8	*Wolfiporia cocos*	8.5 ± 0.0	8.5 ± 0.0

a The initial piece of mycelium had a diameter of 0.8 cm.

The growth of mycelium is significantly influenced by the availability of suitable carbon (C) and nitrogen (N) sources. On the one hand, inorganic N sources, such as nitrate and ammonium sulfate, and organic sources like yeast, malt extract, or peptone may be used, and often combinations of these sources are beneficial.^16,17^ On the other hand, the ratio of the carbon to nitrogen source (C:N ratio) must be carefully managed, as the nitrogen content directly affects the protein content of the mycelium. C:N ratios between 5:1 and 25:1 are considered optimal for fungi.^18,19^ If side streams are used, the use of nitrogen and mineral supplementation may be required to achieve optimal results.^18^ The C:N ratios of BCM (30:1) and OCM (26:1) are close to this optimal range, which explains the fast growth of most fungi. A reason for the worse growth observed on BCA compared to OCA could be attributed to the lower pH (3.6 versus 4.3) as well as the higher content of citric acid (8.2 compared to 4.7 g 100 g^–1^). Citric acid is known to have inhibitory effects on certain fungi.^20^

Besides the carrot side streams used in this study, data for various side streams upcycled with fungi are available. Examples include, but are not limited to, apple pomace, brewer’s spent grain (extract), and wine distillery effluent. While these side streams, along with those used in this study, perform well as sole carbon sources, other side streams such as pomegranate or aronia pomace, leaf spinach, and beet molasses are not suitable as sole carbon sources.^11,21^

### 3.2 Submerged Screening

Twenty-two fungal strains were screened in OCM and 17 fungal strains in BCM. High DM (BCM > 5 g L^–1^, OCM > 10 g L^–1^) in both side-stream media were determined for *Meripilus giganteus II* (MGI), *P. djamor* (PDJ), two different *Pleurotus ostreatus* (POS) strains, *Mycetinis scorodonius* (MSC), and PGE. Furthermore, high CP contents (>20 g 100 g^–1^) were observed for *Fistulina hepatica* (FHE), *Laetiporus persicinus* (LPER), and *Laetiporus sulphureus* (LSU) in OCM and for POS, LPER, and PDJ in BCM (Figure 1). The submerged cultivation was stopped after 10 days in this study, as a decline of DM was observed, suggesting a beginning autolysis of the mycelia, which can have different effects on the product: I) it can lead to the degradation of different cellular products, for example, lactic and citric acid, leading to changes in quality and consistency; II) those degradations might release enzymes such as chitosanases that may affect shelf life; III) it alters sensory properties as shown by Xu et al.^22,23^

**Figure 1:**
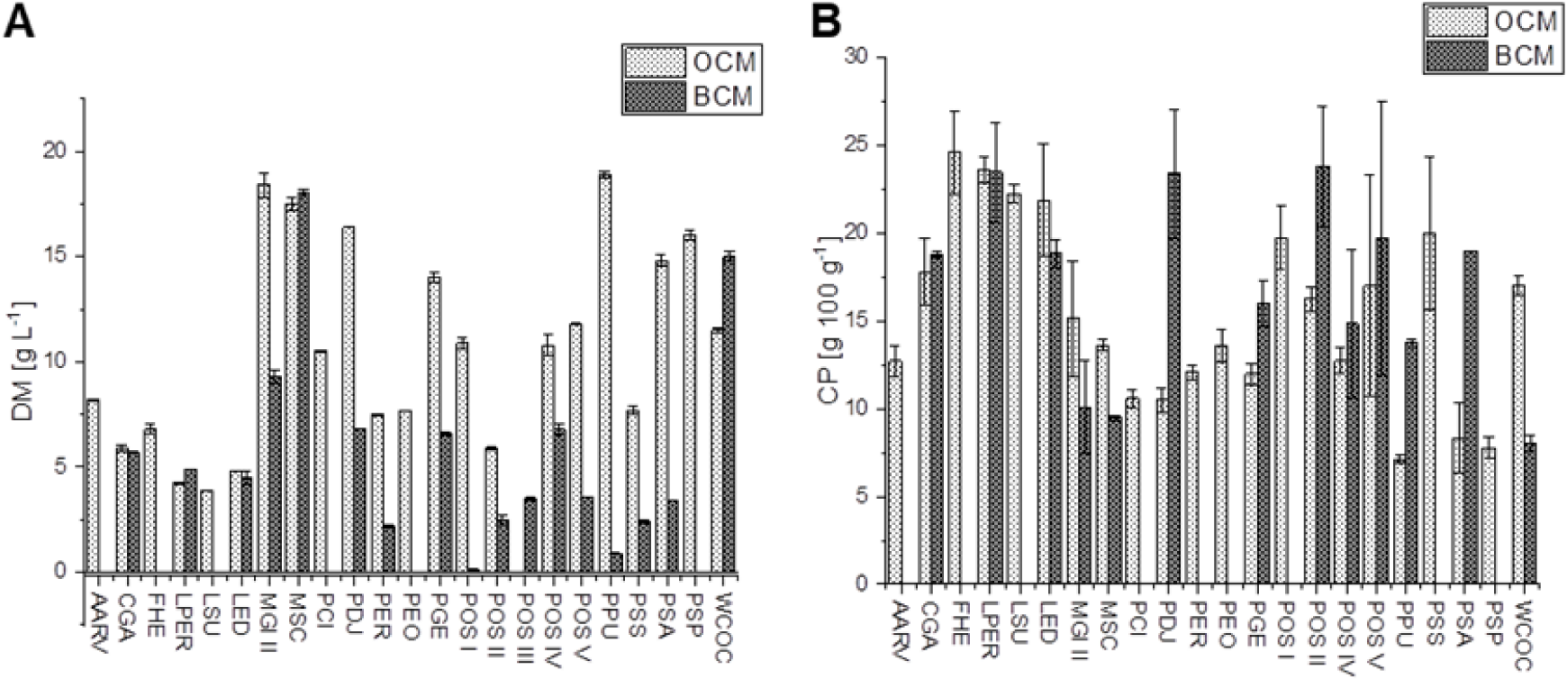
A: Dry matter (DM) content of submerged screened mycelia in 2.2% carbohydrates in orange carrot medium (OCM) and 1.8% carbohydrates in black carrot medium (BCM) (left). B: Crude protein (CP) content, calculated with the Kjeldahl factor of 4.5, of submerged screened mycelia in the OCM and BCM (right). AARV = *Agaricus arvensis*, CGA *=* *Calocybe gambosa*, FHE = *Fistulina hepatica*, LPER = *Laetiporus persicinus*, LSU = *Laetiporus sulphureus*, LED = *Lentinula edodes*, MGI = *Meripilus giganteus*, MSC = *Mycetinis scorodonius*, PCI = *Pleurotus citrinopileatus*, PDJ = *Pleurotus djamor*, PER = *Pleurotus eryngii*, PEO = *Pleurotus sajor-caju*, PGE = *Pleurotus geesterani*, POS = *Pleurotus ostreatus*, PPU = *Pleurotus pulmonarius*, PSS = *Pleurotus salmoneo-stramineus*, PSA = *Pleurotus sapidus*, PSP = *Pleurotus spodoleucus*, WCOC = *Wolfiporia cocos*. Latin numbers are used to distinguish the different isolates of the same species. *n* = 2.

Badalyan et al. studied the growth of different *Pleurotus* spp. on a malt extract medium. After growth for 7 days, they observed DM ranging from 6.5 ± 0.3 for *Pleurotus eryngii* to 29.5 ± 0.3 g L^–1^ for POS, and after 14 days from 12.2 ± 0.2 (*P. eryngii*) to 40.5 ± 0.3 g L^–1^ (POS).^24^ Ahlborn et al. reported fast growth of *Pleurotus salmoneo-stramineus* and *Pleurotus sapidus* on apple pomace. The DM values were 11.7 ± 0.1 and 14.5 ± 0.2 g L^–1^, after only 3 and 4 days, respectively. In the same study, *Wolfiporia cocos* showed a rather slow growth with a final DM of 13.2 ± 0.9 g L^–1^ and a CP of 9.6 ± 0.1 g 100 g^–1^ after 26 days. Overall, DM values ranging from 9.6 ± 0.1 to 15.3 g L^–1^ and CP values ranging from 9.6 ± 0.1 to 25.4 ± 0.3 g 100 g^–1^ were reported, which aligns closely with DM and CP values of this study. Unlike the media used in this study, apple pomace is not fully soluble, and part of the DM was still derived from the nonconverted side stream.^11^ Pilafidis et al. reported yields of 23.8 ± 1.9 g L^–1^ DM with a protein content of 26.5 ± 1.7 g 100 g^–1^ for *P. ostreatus* grown on brewer’s spent grain extract after a cultivation time of 25 days at 26 °C. When grown on diluted wine distillery effluents, the DM was 8.6 ± 0.2 g L^–1^ with a CP of 24.8 ± 1.7 g 100 g^–1^.^21^ While the growth period was about three times longer than the one used in this study, the DM was either lower or similar to those found for the OCM and BCM, with similar CP contents.

### 3.3 Optimization by DoE

Various factors influence the growth of fungal mycelium, which can be categorized into three categories: I) Physical factors, including temperature and stirring speed; II) Chemical factors, such as pH, substrate concentration, and dissolved oxygen saturation; and III) Biological factors, such as inoculum age, volume, and morphology.^25,26^ RSM provides a powerful tool for analyzing the behavior of the data space. It generates a wealth of information from a comparatively low number of experiments, including potential interactions between variables and multiple simultaneous responses, which simplifies the optimization of the process.^14,15^

For optimization of the culture medium, five fungi per side stream were selected: for black carrot side-stream media, two POS (V and VI), PDJ, PGE, and MGI II; and for orange carrot side-stream media, *Agaricus arvensis* (AARV), PDJ, POS VI, PGE, and *Pleurotus spodoleucus* (PSP). All combinations were chosen based on their high DM and/or CP. Using the DoE methodology, response surfaces were generated to determine the optimal CH content and pH value. An example of the generated response surfaces from the cultivations of PGE in different black carrot side-stream media is shown in Figure 2, while the other response surface diagrams are shown in Figures S1–S9 with the relevant statistic data in Tables S5–S24 (Supporting Information).

**Figure 2:**
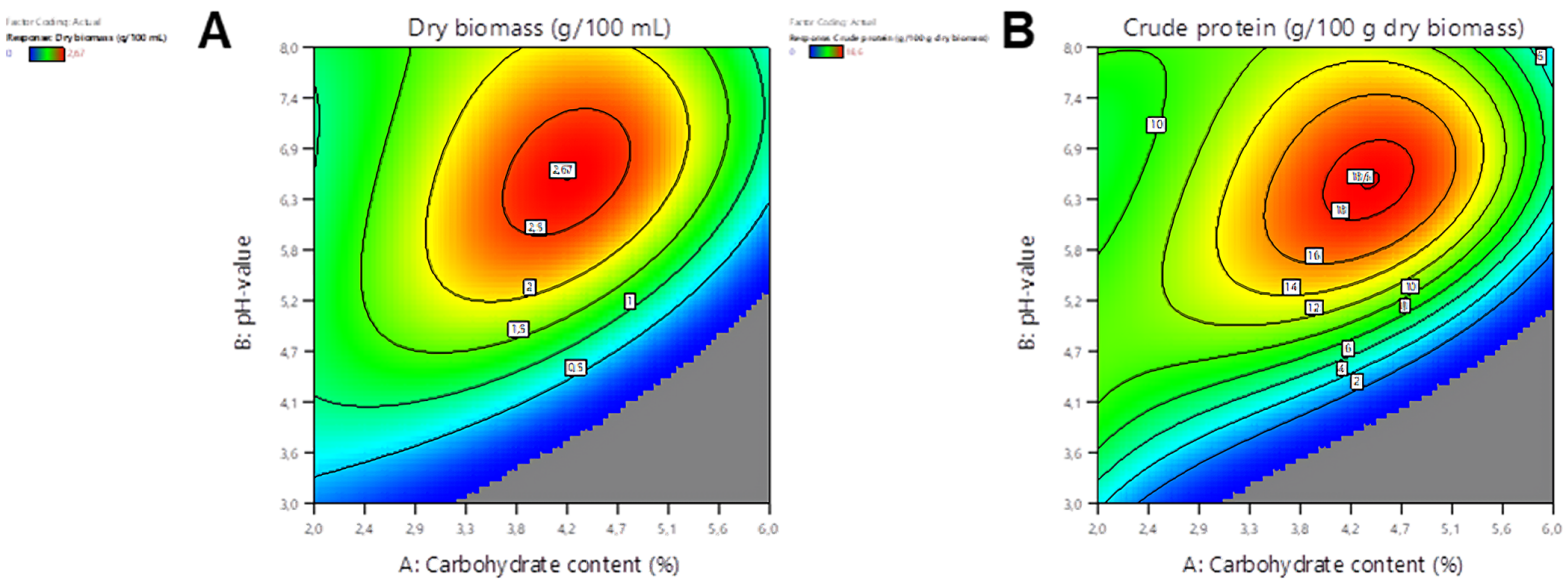
Response surface plot for the dry matter (A) and crude protein contents (Kjeldahl factor: 4.5) (B) of *Pleurotus djamor* grown in black carrot media.

After optimization of the media in terms of CH content and pH value, both DM and CP content showed improvement compared to the initial screening, while the same Kjeldahl factor was used for better comparison. Although the DM increased, the calculated maximum values were not reached. No fungus showed good growth at pH values below 3, and only two fungi, PDJ and PSP, were able to achieve high DM (>15 g L^–1^) with a pH > 7. CH contents over 2% led to increased DM for *Pleurotus* spp., allowing an optimization compared to the initial screening (CH contents of 1.8%/2.2%).

The highest increase in DM was observed for PDJ and POS VI, with factors of 2.6 and 2.0, respectively, in BCO. In OCO, both strains achieved DM yields with an increase of 1.4. In contrast, the estimated maximum CP content was exceeded for almost all of the fungi. In the OCO, every fungus outperformed its screening values, with PSP achieving the most notable improvement, reaching a CP increase factor of 2.1. In BCO, MGI II demonstrated a CP increase by a factor of 1.5, while no increase was found for POS V, POS VI, and PDJ (Figure 3).

**Figure 3:**
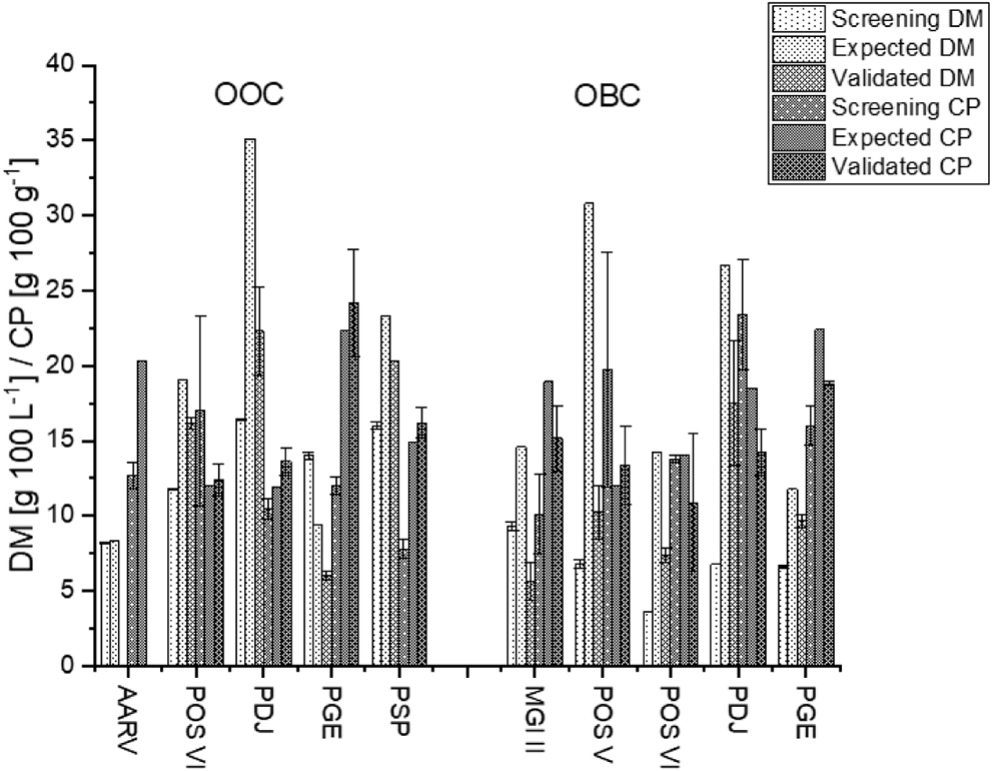
Dry matter (DM) and crude protein contents (CP) of the screening, the expected DM and CP calculated with a Kjeldahl factor of 4.5 of the Design of Experiment and the validated DM and CP of all fungus-medium combinations. OOC = Optimized orange carrot medium for specific fungi, OBC = Optimized black carrot medium for specific fungi, POS = *Pleurotus ostreatus*, AARV = *Agaricus arvensis*, MGI = *Meripilus giganteus II*, PGE = *Pleurotus geesterani*, PSP = *Pleurotus spodoleucus*, PDJ = *Pleurotus djamor*. Latin numbers are used to distinguish different isolates of the same species.

Both pH and CH contents of the medium had a huge impact on the DM and the CP contents. Although fungi are able to grow over a broad pH range, the pH still affects the growth by influencing cell morphology, cell membrane functionality, and enzyme activity.^25,26^ In addition, it was demonstrated that the pH correlates with the CH consumption rate, thereby affecting the production of fungal mycelia.^27^ Other studies have reported that the optimal pH range for the growth of *Pleurotus* spp. is 5–6.^28,29^ In this study, the fungi achieved their highest DM in the pH range from 4.1 to 6.0, consistent with the literature. Bakratsas et al. reported that *P. ostreatus* synthesized more protein at lower initial pH.^30^ However, this effect was not observed in our study.

The initial pH of the OCO medium (4.80 ± 0.01) when fermented with PDJ increased slightly to 4.95 ± 0.01 on day 3 and stabilized at 4.94 ± 0.01 by the conclusion of cultivation on day 6. During the cultivation of PDJ in BCO, notable changes in pH were observed, likely attributable to metabolic by-products. The cultivation began with an initial pH of 6.60 ± 0.02, exhibited a pronounced decline to 5.50 ± 0.16 on day 5 and further decreased to 5.30 ± 0.26 on the final day of cultivation (day 10). These pH shifts are consistent with the known metabolic activity of PDJ, wherein the production of low amounts of organic acids contributes to the acidification of the growth medium.^34^ The distinct pH trends in OCO and BCO suggest substrate-specific variations in PDJ’s metabolic efficiency. When evaluating the influence of the CH content, it is important to note that the used side streams are complex media, meaning that altering the CH content also affects other parameters such as, but not limited to, minerals and nitrogen contents. Different species of *Pleurotus* and *Agaricus* exhibit varying glucose consumption rates during growth.^31^ For example, POS showed a better growth when the glucose concentration was increased from 2% to 4% and *Pleurotus albidus* gained the highest dry matter at 3% sucrose.^26,30,32^ Overall, lower CH (<2%) concentrations resulted in reduced growth, whereas higher concentrations resulted in higher DM, which was consistent with our findings.^26,33^

Bakratsas et al. studied the influence of glucose on the CP content of POS using a range from 0.5% to 8.0%. They found that CH contents below 1% resulted in a low CP content, while the highest CP content was achieved at a concentration of 2%. Therefore, they concluded that higher concentrations led to a reduction of protein synthesis.^30^ A similar trend was observed for three different combinations of POS and side-stream media evaluated in this study.

### 3.4 Nutritional Composition of *Pleurotus djamor* Mycelium

PDJ was selected for further studies due to I) its good growth on both side streams, II) its neutral olfactory profile, and III) its high market potential within the *Pleurotus* spp. family. Additionally, when comparing the DM formed in BCO, OCO, and malt extract, BCO (17.5 ± 4.5 g L^–1^) and OCO (22.0 ± 3.0 g L^–1^) resulted in 2.3- and 3.0-fold higher DM yield than in malt extract (7.7 ± 0.0 g L^–1^) on day 7. Furthermore, the CP in the mycelium cultivated in the side streams reached 13.6 ± 0.9 g 100 g^–1^ (OCO) and 14.2 ± 1.6 g 100 g^–1^ (BCO), respectively. The CP of the PDJ mycelium in malt extract (14.5 ± 0.6 g 100 g^–1^) was in a similar range to the mycelium cultivated in the side streams. However, when considering the increased biomass yield from the side streams, the overall CP productivity per liter of medium in malt extract medium is 3.0-fold lower: 1.1 ± 0.0 g CP L^–1^ in malt extract medium versus 3.0 ± 0.6 g CP L^–1^ in OCO. These results highlight the promising potential of OCO as a cost-effective cultivation medium, offering comparable crude protein yields at a fraction of the cost of malt extract, which is priced at approximately 7.5 € kg^–1^ (187 € per 25 kg by Mr. Malt).

The nutritional composition of mycelia is dependent on the fungus and the substrate used. After the optimization of the biomass and crude protein production, the nutritional composition of the PDJ mycelium as a promising protein source was analyzed (Table 2). Therefore, PDJ cultivation was scaled up to 2 L flasks in both optimized side-stream media. This cultivation resulted in DM yields of 15.5 ± 2.7 g L^–1^ for the OCO and 13.7 ± 2.3 g L^–1^ for the BCO.

**Table 2:** Composition of the Mycelium of *Pleurotus djamor* Grown in Optimized Black Carrot Medium (OCO) and the Optimized Black Carrot Medium (BCO)^a^

Content in [g·100 g^–1^]	OCO	BCO
**CP**	31.0 ± 5.9	21.6 ± 1.9
**Ash**	5.9 ± 0.8	7.1 ± 0.9
**Fat**	1.7 ± 0.0	2.4 ± 0.2
**Glucans**	14.1 ± 6.2	19.2 ± 4.0
**α-Glucan**	9.5 ± 3.5	15.6 ± 2.7
**β-Glucan**	4.6 ± 2.7	3.6 ± 1.3
**Chitin**	4.1 ± 0.0	5.4 ± 0.0
**Total sugars**	18.5 ± 3.8	6.6 ± 1.1
**Reducing sugars**	15.9 ± 3.3	6.6 ± 1.1
**Sucrose**	2.6 ± 0.5	-

a Crude protein (CP) was calculated with the specific Kjeldahl factor based on the amino acid analysis.

The CP content of mycelia ranges from 19 to 40 g 100 g^–1^.^35,36^ The PDJ mycelia contained 21.6 ± 1.9 g 100 g^–1^ and 31.0 ± 5.9 g 100 g^–1^ CP for BCO and OCO, respectively. Manu-Tawiah and Martin cultivated *P. ostreatus* in two different media: a synthetic medium and a medium containing peat extract. While the POS cultivated in the peat extract medium reached a CP content of 40.1 ± 1.8 g 100 g^–1^, only 25.7 ± 1.8 g 100 g^–1^ were obtained in the synthetic medium.^35^ For PDJ, a high difference of about 30% in CP, when cultivated in OCO and BCO, was found as well. Since the crude protein content accounts for more than 20% of the caloric value of the mycelium, it can be considered high in protein.^37^

The sulfur-containing amino acids methionine and cysteine are often the limiting amino acids of fungi; this was also the case for PDJ (together with tryptophan) cultivated in both optimized side-stream media (as shown in Figure 4), and glutamine/glutamic acid and asparagine/aspartic acid were predominantly present. The biological value (BV) of the mycelium of PDJ on BCO was 83 ± 1, and 19% higher than that in OCM (68 ± 0). While the BV of the mycelium grown on BCO is similar to those of most animal-derived proteins (77 for casein, 80 for beef, and 91 for milk), the BV of the mycelium grown on OCO is similar to most plant-based proteins, such as soy (74) and wheat gluten (64).^38^ To gain higher BV, a combination with other protein sources, for example cereals, where usually lysine and threonine are limiting, is possible.^39^

**Figure 4:**
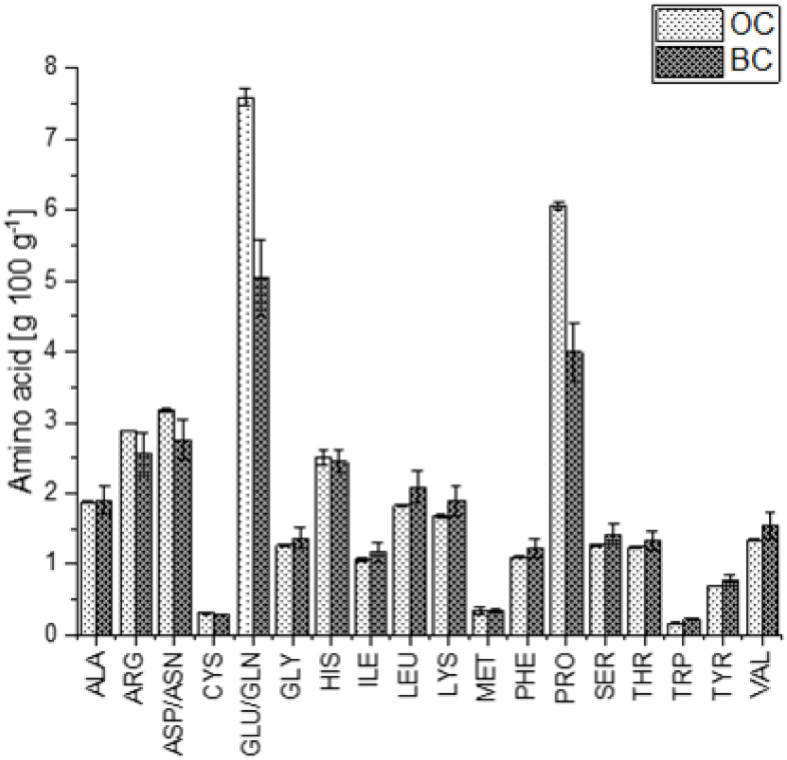
Amino acid profile of the mycelium of *Pleurotus djamor* in the optimized orange carrot medium (OCO) and the optimized black carrot medium (BCO). *n* = 2.

Regarding the fiber content, mycelia contain two main fractions: chitin and glucans, both of which are components of the fungal cell wall.^40^ Consequently, mycelia contain high concentrations of chitin and glucans, while no chitin is present in the side-stream-based media. The chitin content of the mycelium of PDJ ranged from 4.1 ± 0.0 (OCO) to 5.4 ± 0.0 g 100 g^–1^ (BCO), aligning with other Basidiomycota such as *Pleurotus sapidus* (PSA) grown on apple pomace with 6.3 ± 0.4 g 100 g^–1^ and *Pleurotus tuber-regium* grown on a synthetic medium (3.3–6.8 g 100 g^–1^).^11,41^ Similar values were reported in a study of Manu-Tawiah and Martin were the differences in fat and fiber contents were minor, with fat contents of 3.7 ± 0.4 (peat) and 3.0 ± 0.3 g 100 g^–1^ (synthetic), and fiber contents of 5.9 ± 0.5 (peat) and 5.0 ± 0.3 g 100 g^–1^ (synthetic).^35^ Mycelia are not only high in protein but are also known to contain little fat. Compared to the mycelium of POS, the mycelium of PDJ contained with 1.7 ± 0.0 g 100 g^–1^ (OCO) and 2.4 ± 0.2 g 100 g^–1^ (BCO) even less fat.^35^

The total glucan contents were 19.2 ± 4.0 g 100 g^–1^ (BCO) and 14.0 ± 6.1 g 100 g^–1^ (OCO). Interestingly, unlike most fungi, the PDJ mycelium displayed a higher α-glucan content compared to β-glucans. While the β-glucan content ranged from 3.6 ± 1.3 (BCO) to 4.6 ± 2.7 g 100 g^–1^ (OCO), the α-glucan content was significantly higher, ranging from 9.5 ± 3.5 (OCO) to 15.6 ± 2.7 g 100 g^–1^ (BCO). For *Lentinula edodes*, the α-glucan content ranged from 1.5 ± 0.1 to 7.9 ± 0.3 g 100 g^–1^ and the β-glucan content from 15.6 ± 6.0 to 27.1 ± 2.1 g 100 g^–1^, resulting in total glucan contents of 22.5 ± 3.9 to 34.9 ± 2.1 g 100 g^–1^.^42^ The total glucan content of PDJ mycelium (22.0 ± 0.7 and 26.9 ± 2.8 g 100 g^–1^) was comparable, while the share of α-glucans was 56% (OCO) and 85% (BCO) of the total glucan content.

### 3.5 Sensory Evaluation of the Mycelium-Based Products and Their Acceptance

The patties were described as pink to brownish, closely resembling the appearance of the minced meat. The patty with 100% soy protein isolate was perceived as firmer, drier, and having a floury texture. In contrast, as the proportion of mycelium increased, the patties became softer and crumblier (Figure 5).

**Figure 5:**
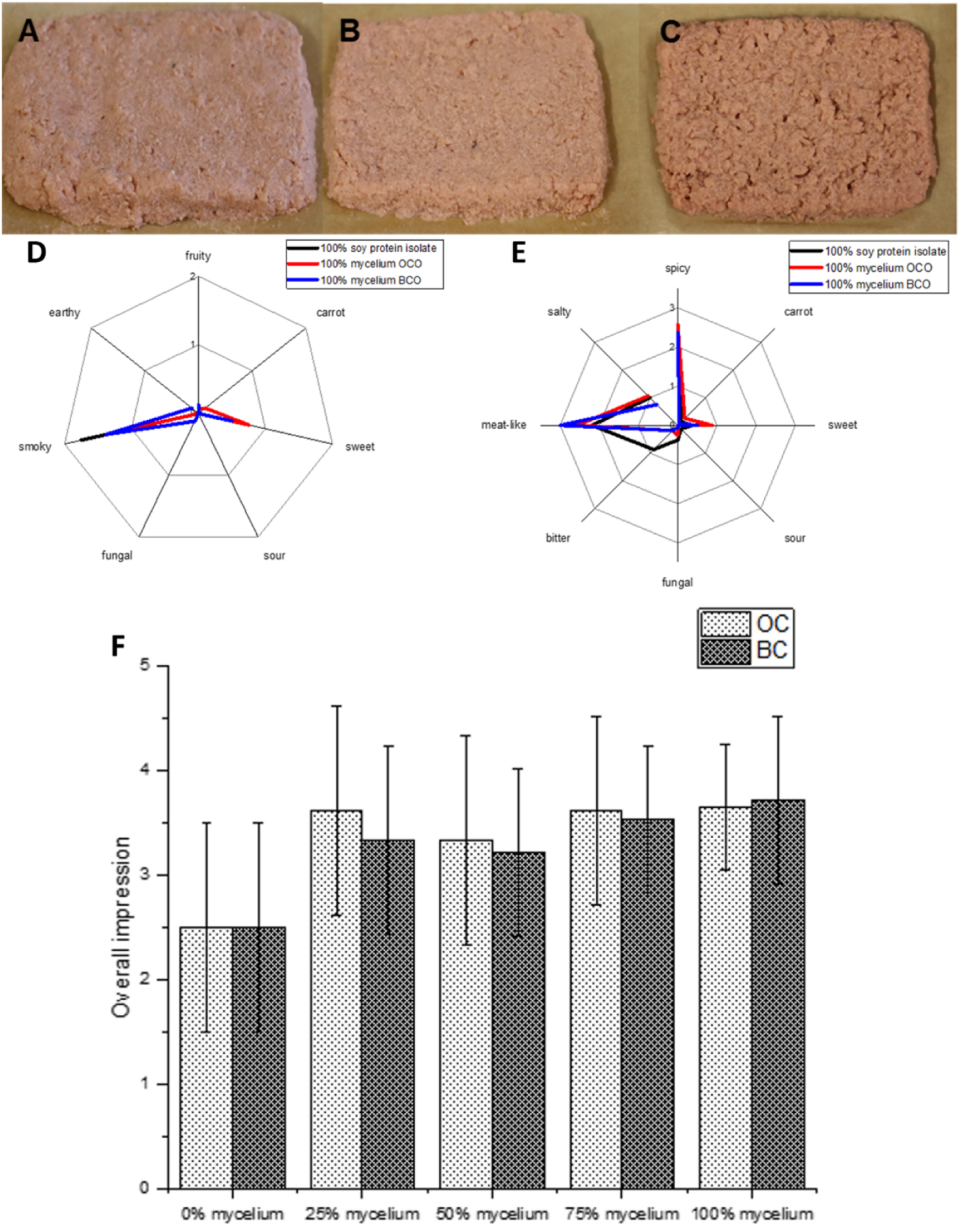
A: 100% soy protein isolate patty; B: 25% mycelium and 75% soy protein isolate patty; C: 100% mycelium. Sensory profiles of the smell (D) and taste (E) of the burger patties made of soy protein isolate as well as the mycelium of *Pleurotus djamor* grown on the optimized orange carrot medium (OCO) and the optimized black carrot medium (BCO). F: The overall impression, rated from 0 (not good) to 5 (very good), of the burger patties was a result of varying amounts of mycelium replacements (0% mycelium indicates 100% soy protein isolate, while 100% mycelium indicates 0% soy protein isolate).

Olfactory and gustatory attributes across the different patties exhibited similar profiles. The main olfactory descriptors included an average intensity of smoky, oily, and toasty notes, accompanied by a subtle sweet odor. The taste was described as spicy and meat-like, with a slight sweetness. Patties containing 100% soy protein isolate were noted to have a bitter aftertaste.

The patties with 100% mycelium as a replacement for soy protein isolate received the highest overall likeness rating of 3.5 out of 5, while those containing 100% soy protein isolate were rated the lowest with 2.5. No significant difference (*p* > 0.05) could be found for the acceptance between patties made with the mycelium of orange and black carrot, but a significant difference (*p* < 0.05) was found between the mycelium and soy patties leading to a superior overall evaluation of the mycelium-based patties. This was largely attributed to their texture, as olfactory and gustatory differences were minor.

The sausages containing mycelium showed a darker color compared to the yellowish color of the sausages containing chickpeas (Figure 6). All sausages showed a spongy, firm texture.

**Figure 6:**
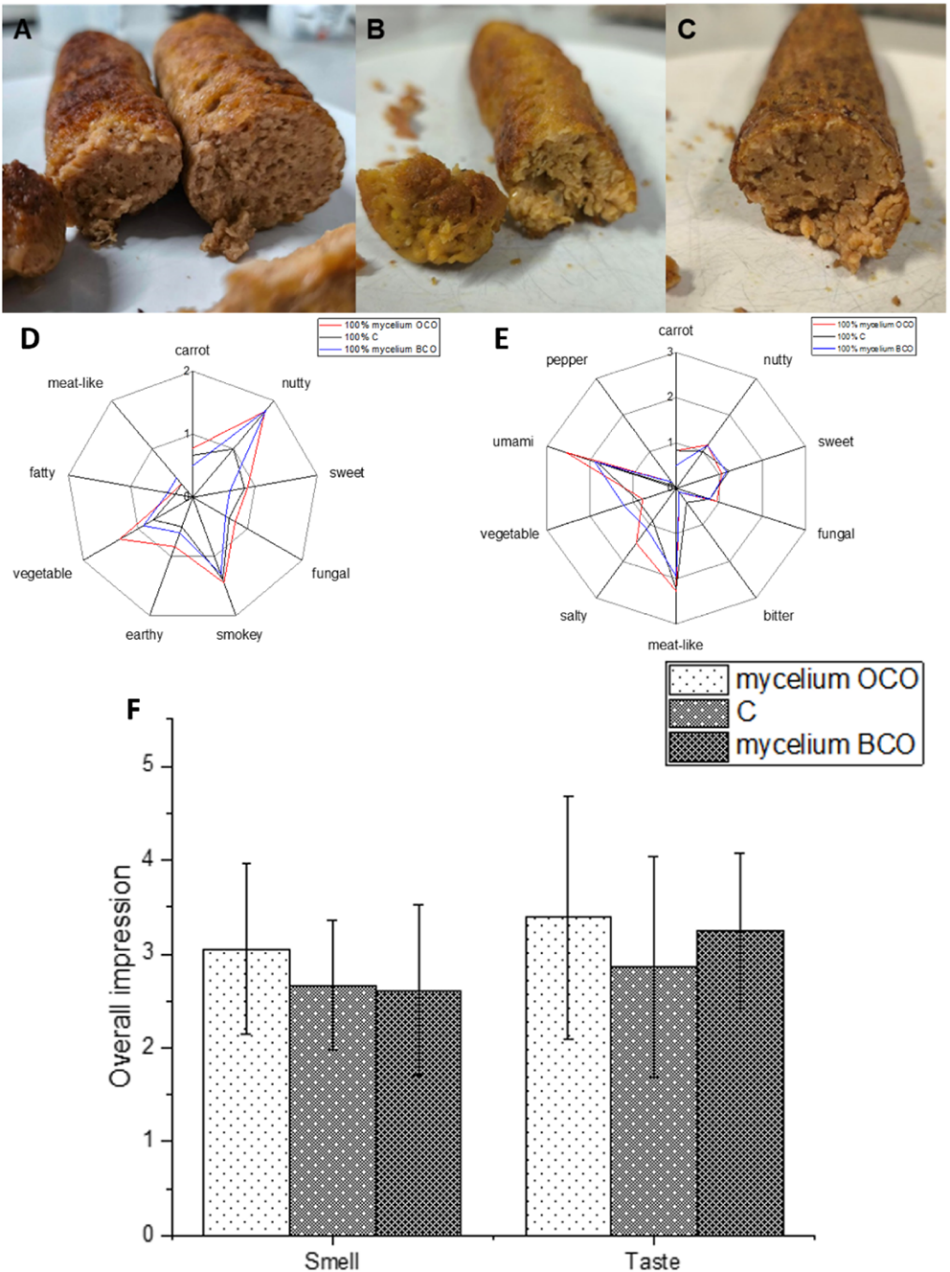
A: Sausage with 100% *Pleurotus djamor* mycelium (PDJ) grown in the optimized orange carrot medium (OCO). B: Sausage with 100% chickpeas (C). C: Sausage with 100% PDJ grown in the optimized black carrot medium (BCO) (right). Sensory profiles of the smell (D) and taste (E) of the sausages made of chickpeas and mycelium. F: Liking of smell and taste of the sausages rated from 0 (not good) to 5 (very good).

Olfactory and gustatory attributes across the different sausages showed similar profiles, and no significant differences were identified for both smell and taste (*p* > 0.05). All sausages were described to smell nutty, smoky, and vegetable-like in different degrees. OCO was the most intense, while that of BCO was the least intense one. A sweet smell was noted for both mycelium-based sausages, while this note was far less intense in the chickpea-based sausage. All sausages were described to taste meat-like as well as umami. The highest intensity was found for the sausages made of mycelium grown on an OCO. While the chickpea-based sausages had a slightly bitter off-flavor, this was far less intense for the mycelium-based sausages.

Studies by Kim et al. found that burger patties made with the mycelium of *Agaricus bisporus* were more favorably received than those made with soybeans.^8^ Furthermore, a study based on the use of mycelium of PSA in a vegan boiled sausage analog received higher taste ratings compared to a vegan alternative.^43^ These results, as well as the results of our study, highlight mycelium as a promising meat alternative with appealing sensory properties.

Further supporting evidence comes from Zhang et al., who demonstrated that adding mycelium to a soy-based meat analog leads to an increased umami taste of the product and reduces the beany off-flavor, making its flavor closer to that of meat.^44^ Furthermore, they showed that a mycelium content of less than 8% can suppress bitterness as well as the astringency of the patties. The addition of mycelium positively influenced the color and the texture of the meat analogs, making them springier, brighter, and whiter, while redness was reduced compared to the 100% soy protein meat analog.^44^ These findings align with those of Kim et al., who claimed that meat analogs with mycelium are higher in hardness, springiness, and chewiness, resulting in improved textural properties.^8^ These properties could be one reason why the panel in the present study favored the 100% mycelium patties over those made with soy protein isolate, despite minimal differences in terms of taste. Still, stability over time must be investigated for those products. For example, Stephan et al. further observed a similar hardness of sausages based on soy protein and mycelium, reinforcing the value of mycelium in meat analog applications.^43^

After screening of over 100 fungal strains in two side-stream-based media, a strain, PDJ, was identified, whose mycelium exhibited a neutral taste and aroma in both media. By employing an experimental design approach, the medium composition was optimized, achieving significantly higher DM yields. Remarkably, the mycelium grown in carrot side-stream media is high in protein and contains high α-glucan and β-glucan levels.

When the mycelium was used for the preparation of meat analogs, the mycelium-based patties outperformed soy protein isolate-based patties in sensory evaluation. These findings suggest that PDJ mycelium cultivated on liquid carrot side streams from the production of natural colorants represents a sustainable, high-quality alternative to traditional plant-based meat analogs, offering promising sensory and nutritional properties while making efficient use of food industry by-products.

By utilizing these side streams as substrate, the mycelium production not only reduces environmental impact but also adds significant value to these by-products. This approach demonstrates a high potential for addressing food security issues, as it enables the efficient production of high-quality, sustainable protein sources. Consequently, the submerged fermentation of mycelium from edible fungi could play a crucial role in feeding a growing global population, while promoting a more circular and waste-reducing food system.

## Supplementary Material



## Notes

Table S1: All fungi used for the screening in surface cultures, their original source, the cultivation medium for strain maintenance, and the sensory evaluation on orange carrot agar (OCA) and black carrot agar (BCA). Table S2: Nutritional composition of the side streams of black carrot (BC) and orange carrot (OC). Table S3: All fungi screened in submerged culture in 2.2% carbohydrate orange carrot medium (OCM) and 1.8% carbohydrate black carrot medium (BCM), and the day with their highest dry matter (DM). Table S4: Design of Experiment for the optimization of the submerged culture on black carrot medium and orange carrot medium. CH: carbohydrate; DM: dry matter; CP: crude protein. Tables S5–S24: Statistic values of the Design of Experiment for dry matter or the crude protein of the different fungi in the orange or black carrot media. Figure S1: Response surface plot for AARV on OCM for dry biomass (left) and crude protein content (right). Figure S2: Response surface plot for PDJ on OCM for dry biomass (left) and crude protein content (right). Figure S3: Response surface plot for PGE on OCM for dry biomass (left) and crude protein content (right). Figure S4: Response surface plot for POS VI on OCM for dry biomass (left) and crude protein content (right). Figure S5: Response surface plot for PSP on OCM for dry biomass (left) and crude protein content (right). Figure S6: Response surface plot for MGI II on BCM for dry biomass (left) and crude protein content (right). Figure S7: Response surface plot for POS V on BCM for dry biomass (left) and crude protein content (right). Figure S8: Response surface plot for POS VI on BCM for dry biomass (left) and crude protein content (right). Figure S9: Response surface plot for PGE on BCM for dry biomass (left) and crude protein content (right) (PDF)

## Abbreviations

AARV: *Agaricus arvensis*
BC: black carrot side stream
BCA: black carrot agar
BCM: black carrot medium
BCO: optimized black carrot medium
BV: biological value
CGA: *Calocybe gambosa*
CH: carbohydrate
CP: crude protein
DoE: design of experiment
DM: dry matter
FHE: *Fistulina hepatica*
LPER: *Laetiporus persicinus*
LSU: *Laetiporus sulphureus*
LED: *Lentinula edodes*
MGI: *Meripilus giganteus*
MSC: *Mycetinis scorodonius*
OBC: optimized black carrot medium for specific fungi
OC: orange carrot side stream
OCA: orange carrot agar
OCM: orange carrot medium
OCO: optimized orange carrot medium
OFAT: one-factor-at-a-time
OOC: optimized orange carrot medium for specific fungi
PCI: *Pleurotus citrinopileatus*
PDJ: *Pleurotus djamor*
PEO: *Pleurotus sajor-caju*
PER: *Pleurotus eryngii*
PGE: *Pleurotus geesterani*
POS: *Pleurotus ostreatus*
PPU: *Pleurotus pulmonarius*
PSS: *Pleurotus salmoneo-stramineus*
PSA: *Pleurotus sapidus*
PSP: *Pleurotus spodoleucus*
RSM: response surface methodology
WCOC: *Wolfiporia cocos*
