# Supplementary material for: Pleurotus djamor Mycelium: Sustainable Production of a Promising Protein Source from Carrot Side Streams
Source: J Agric Food Chem. 2025 Dec 17;73(52):33156–66. doi: 10.1021/acs.jafc.5c11223 (PMC12766720; doi:10.1021/acs.jafc.5c11223)
Supplement: Supplementary file 1 [file jf5c11223_si_001.pdf]

Supporting information

Pleurotus djamor mycelium: sustainable production of a promising protein source from carrot side streams

Leonie Cora Juhrich[a], Iris Lammersdorf[a], Pascal Schmitt[a], Lars Tasto[b], Falk Speer[c], Denise Salzig[b], Kai Reineke[c], Holger Zorn[a,d], Martin Gand[a,*]

[a] Institute of Food Chemistry and Food Biotechnology, Justus Liebig University Giessen, Heinrich-Buff-Ring 17, 35392 Giessen, Germany

[b] Institute of Bioprocess Engineering and Pharmaceutical Technology, University of Applied Sciences Middle Hesse, Gutfleischstrasse 3-5, 35390 Giessen, Germany

[c] GNT Europa GmbH, Kackertstrasse 22, 52072 Aachen, Germany

[d] Fraunhofer Institute for Molecular Biology and Applied, Ecology, Ohlebergsweg 12, 35392 Giessen, Germany

Detailed description of the methods

To determine the crude protein content (CP), the Kjeldahl-method with 0.2 g lyophilized mycelium, 25 mL concentrated sulfuric acid, and 2 catalysator tablets was used. The digestion was performed by the Turbotherm (C. Gerhardt GmbH & Co. KG, Königswinter, Germany). Afterward, a steam distillation with 90 mL water and 80 mL 32-33% NaOH was performed by the Vapodest 450 (C. Gerhardt GmbH & Co. KG) into an 80 mL boric acid condenser. An automatic titration with 0.1 M HCl followed. The CP content was calculated using formula S1.

$$CP = \frac{1.4007 \cdot (V - V_{\mathrm{B}}) \cdot F}{10 \cdot m_{\mathrm{mycelium}}} \qquad \text{(S1)}$$

CP:	crude protein content [g 100 g^{-1}]
V:	volume of 0.1 M HCl for sample [mL]
V_B:	volume of 0.1 M HCl for blank [g]
F:	nitrogen to protein conversion factor (4.50 for screening and optimization, 5.96 for PDJ in orange carrot media and 5.83 for PDJ in black carrot media)
$m_{mycelium}$:	mass of mycelium [g]

The ash content was measured by incinerating the mycelium sample using a Bunsen burner and ashed in a muffle oven (Nabertherm GmbH, Lilienthal, Germany) at 550 °C and comparing the residual weight with the original weight of the used jar based on formula S2.

$$A = \frac{(m_{\mathrm{ash}} - m_{\mathrm{empty}})}{m_{\mathrm{mycelium}}} \cdot 100 \qquad \text{(S2)}$$

A:	ash content [g 100 g^{-1}]
m_{ash}:	ash in jar [g]
m_{emptry}:	empty jar [g]
m_{mycelium}:	sample [g]

The fat content was determined by the method of Weibull-Stoldt. About 2 g of the dry, ground mycelium was weighed on fat free paper and was transferred into the fusion flasks for the Hydrotherm (C. Gerhardt GmbH & Co. KG) device. The fusion was under hydrochloric

conditions with 18 cycles. The filter paper together with the lipids was dried at 105 °C for 2.5 h. It was transferred into filtration shells and closed with cotton. The filtration flasks were weighed before together with boiling chips. Afterward, the lipid was extracted with petroleum ether using a soxtherm-extraction device (C. Gerhardt GmbH & Co. KG). The flask was dried at 105 °C and weighed until the weight stability. Formula S3 was used to calculate the fat content.

$$F = \frac{m_{\text{lipid}} - m_{\text{empty}}}{m_{\text{mycelium}}} \cdot 100 \qquad \text{(S3)}$$

F:	fat content [g 100 g^{-1}]
m_{lipid}:	flasks with extracted lipid [g]
m_{empty}:	empty flask [g]
m_{mycelium}:	sample [g]

To determine the chitin content, formula S4 was used.

$$E = 0.0231 \text{ L mg}^{-1} \cdot \beta + 0.0459 \text{ mg L}^{-1} \qquad \text{(S4)}$$

E:	extinction at 650 nm
β:	concentration of *N*-acetylglucosamine [mg L^{-1}]

For the analysis of amino acids, the amino acid analyzer S433 (Sykam GmbH) with columns LCA K13/Na and LCA K04/ Na S433 (Sykam GmbH) and Chromstar software v. 7 (Sykam GmbH) were used. 25 mg dry, ground mycelium was used for each sample. For total amino acids, 2.5 mL 6 M phenolic HCl was used. For the analysis of cysteine and methionine, 0.5 mL oxidation solution (H_2O_2 and phenolic formic acid (10:90)) was used. Furthermore, 0.084 g sodium metabisulfite was added after 16 h. For tryptophane, 2.5 mL phenolic NaOH was added; after hydrolysis, the pH was adjusted in two steps first to 2.2 with 0.5 M phosphoric acid, followed by the addition of 1 M HCl to 3.75. Specific Kjeldahl factors were calculated using formula S5.

$$F_{\text{spec}} = \frac{A_{\text{res}}}{N_{\text{Protein}}} \qquad \text{(S5)}$$

F_{spec}:	specific Kjeldahl factor
A_{res}:	amino acid residual [g 100 g^{-1}]
N_{protein}:	nitrogen in protein [g 100 g^{-1}]

The sugars in the mycelium were analyzed by thin layer chromatography. 5 g dry, ground mycelium was extracted with 30 mL water and purified by Carrez precipitation as described by Matissek and Fischer 2021.[1] Pre-dried aluminum oxide 60 G plates (Merck KGaA) were used. Glacial acetic acid, dichloromethane and desalted water (35/30/5) were used as mobile phase. After separation, the plate was dried, followed by visualization by spraying a solution of a mixture of 10 g trichloroacetic acid, 5 g phthalic acid and 1.2 g *p*-amino hippuric acid in 200 mL ethanol and incubation for 15 min at 135 °C in a fume hood. Afterward, the plate was sprayed with 15 g urea in 45 mL 2 M hydrochloric acid and the retention factor was determined using formula S6.

$$R_f = \frac{s}{h} \qquad \text{(S6)}$$

R_f: retention factor

s: distance of spot to starting line [cm]

h: solvent front [cm]

The content of reducing sugars was determined using the method of Luff-Schoorl (formula S7). 4 g of dried, ground mycelium was extracted with water, followed by Carrez precipitation, filled in a 250 mL-volumetric flask and filtrated. 25 mL of the filtrate was boiled for 2 min with 25 mL Luff-solution (citric acid monohydrate, sodium carbonate, copper sulfate-pentahydrate) and cooled down with water afterward. 10 mL of potassium iodide-solution (300 g L^{-1}) and 25 mL 25% sulfuric acid were added. Afterward, the formed iodine was titrated with 0.1 M sodium thiosulfate solution. Starch solution was added as indicator.

$$S_{red} = \frac{I \cdot F \cdot 1000\ \text{mL L}^{-1}}{V \cdot 1000\ \text{mg g}^{-1}} \qquad \text{(S7)}$$

S_{red}: content of reducing sugars as inverted sugar [g 100 g^{-1}]

I: sugar in 25 mL aliquot

F: dilution factor

V: volume [mL]

Furthermore, 25 mL of the purified solution was filled ad mark with water and 5 mL of 37% hydrochloric acid in a 100 mL volumetric flask. The solution was heated to 67-70 °C for 5 min. Then it was immediately cooled down to 20 °C. Phenolphthalein was added and the solution with NaOH neutralized. 25 mL of this solution was treated and analyzed as reducing sugars

described before. Total sugar was calculated using formula S8 and sucrose content using formula S9.

$$S = \frac{I \cdot F \cdot 1000\ \text{mL L}^{-1}}{V \cdot 1000\ \text{mg g}^{-1}} \quad \text{(S8)}$$

$$\text{Sucrose} = S - S_{Red} \quad \text{(S9)}$$

Table S1: Fungi used for the surface screening, their original source, the cultivation medium for strain maintenance and the sensory evaluation on orange carrot agar (OCA) and black carrot agar (BCA). MEA = malt extract agar, MEPA = Malt extract pepton agar, Blackwood Forest = kindly provided by Flavius Popa from the Blackwood Forest national park, CBS = Westerdijk Fungal Biodiversity Institute former name Centraalbureau voor Schimmelcultures, Utrecht, Netherlands**,** DSMZ = German Collection of Microorganisms and Cell Cultures GmbH of the Leibniz Institute, Braunschweig, Germany, Fa. Sylvan = company Sylvan Kittanning, Pennsylvania Fraunhofer = Collection of the Fraunhofer Institute for Molecular Biology and Applied Ecology IME, Giessen, Germany, IMD = Institut für Mykologie der Biologischen Bundesanstalt, Berlin, Germany, Göttingen = kindly provided by Prof. Dr. Ursula Kües, LCB = Collection of the Insitute of food chemistry and food biotechnology of the Justus Liebig University, Giessen, Germany, Mycelia = company Mycelia NV, Deinze, Belgium, Steintaler Edelpilze = Steintaler Edelpilze, Neu Wulmstorf, Germany Yoichi Honda = kindly provided by Prof. Dr. Yoichi Honda of the Kyoto University.

Fungus	Source	Medium	OCA sensory	BCA sensory
Abortiporus biennis	CBS	MEA	neutral	sweet, fruity
Agaricus arvensis	CBS	MEA	fungal, chemical	fungal
Agaricus bitorquis	DSMZ	MEPA	egg	sweet, fruity
Amylostereum chailletii	CBS	MEA	sweet, malt	green
Armillaria bulbosa	DSMZ	MEA	fungal	sweet
Armillaria gallica	LCB	MEPA	fungal	sweet
Armillaria mellea	DSMZ	MEA	fungal	neutral
Armillaria tabescens	DSMZ	MEA	sour	sour
Auricularia fuscosuccinea	LCB	MEA	fermented	neutral
Bovista plumbea	Fraunhofer	MEA	neutral	sweet
Calocybe gambosa	LCB	MEA	fungal	sour, smokey
Ceriporiopsis resinascens	CBS	MEA	fungal, smokey	fungal
Clitocybe gibba	LCB	MEA	fruity, sweet	fungal
Clitocybe odora	DSMZ	MEPA	fruity, sweet	sweet
Clitopilus hobsonii	LCB	MEPA	neutral	fermented, sour
Coprinellus flocculosus	LCB	MEPA	neutral	sweet
Coprinus cinereus	Fraunhofer	MEA	neutral	neutral
Coprinus comatus	LCB	MEA	fungal, fruity	sweet
Coprinus erythrocephalus	DSMZ	MEA	fungal	sweet
Coprinus sterquilinus	DSMZ	MEA	green	sweet
Coprinus xanthothrix	DSMZ	MEA	sweet, floral	sweet, fruity

Fungus	Source	Medium	OCA sensory	BCA sensory
Cyathus helenae	CBS	MEA	fruity, sweet	sweet
Cyclocybe aegerita	Fa. Sylvan	MEA	fungal	neutral
Cystostereum murrayi	Blackwood forest	MEPA	sour	fruity, fungal
Exidia glandulosa	DSMZ	MEA	fruity	sour
Fistulina hepatica	DSMZ	MEA	urinal	green
Flammula alnicola	CBS	MEA	fungal	fungal
Flammulina velutipes I	DSMZ	MEPA	green	fruity, green
Flammulina velutipes II	LCB	MEA	green	sour
Flammulina velutipes III	DSMZ	MEA	green	sweet
Fomitopsis betulinus	LCB	MEPA	green, fungal	fungal, fermented
Ganoderma lucidum	DSMZ	MEPA	sweet	fungal
Gloeophyllum abietinum	DSMZ	MEA	sweet, fruity	sweet, fruity
Gloeophyllum odoratum	DSMZ	MEA	sour	fruity, sour
Gloeophyllum sepiarium	LCB	MEPA	fungal, smokey	smokey
Gloeophyllum trabeum	LCB	MEPA	floral	floral, sour
Hericium cirrhatum	CBS	MEA	fruity, sweet	sweet
Hericium coralloides	CBS	MEA	chemical	chlorine
Hericium erinaceus	IMD	MEA	fungal	fruity
Hericium flagellum	Fraunhofer	MEA	neutral	floral, sweet
Hymenopellis radicata	LCB	MEPA	fungal	fungal, green
Hypsizygus tessulatus	DSMZ	MEA	fungal	sour
Kuehneromyces mutabilis I	LCB	MEPA	fungal	fungal, green
Kuehneromyces mutabilis II	DSMZ	MEA	fungal	fungal
Laetiporus persicinus	CBS	MEA	fungal, spicy	smokey, chemical
Laetiporus sulphureus	DSMZ	MEA	spicy	spicy, fungal
Lentinula edodes	CBS	MEA	fungal	sweet
Lepista nuda	DSMZ	MEA	urinal	urinal
Lycoperdon pyriforme I	DSMZ	MEA	sweet, fruity	chemical
Lycoperdon pyriforme II	LCB	MEPA	chemical	sweet, sour
Macrolepiota procera I	Göttingen	MEA	fungal	fungal
Macrolepiota procera II	LCB	MEPA	chemical	fungal
Marasmius alliaceus	LCB	MEA	neutral	sweet, garlic
Meripilus giganteus I	DSMZ	MEPA	sour, fungal	neutral
Meripilus giganteus II	LCB	MEA	fungal	neutral
Merulius tremellosus	DSMZ	MEA	neutral	neutral
Mycena pseudocorticola	LCB	MEPA	fruity, floral	floral
Mycetinis scorodonius	CBS	MEA	garlic	garlic
Neolentinus lepideus	Fraunhofer	MEA	floral	floral, sweet
Phanerochaete chrysosporium	CBS	MEA	fungal	smokey, fermented
Phlebia centrifuga	Blackwood forest	MEA	fungal, sour	sweet
Pholiota lignicola	LCB	MEPA	fungal, floral	fungal, green

Fungus	Source	Medium	OCA sensory	BCA sensory
Pholiota nameko	DSMZ	MEA	fungal	sour
Pleurotus citrinopileatus	DSMZ	MEPA	fungal	sweet
Pleurotus cornucopiae	Göttingen	MEA	fungal	neutral
Pleurotus cornucopiae var. citrinopileatus	Steintaler Edelpilz	MEA	floral	sweet
Pleurotus cystidiosus var.	Steintaler Edelpilz	MEA	neutral	neutral
Pleurotus djamor var.	Steintaler Edelpilz	MEA	neutral	sweet
Pleurotus dryinus	DSMZ	MEPA	fungal	sweet, sour
Pleurotus eryngii	DSMZ	MEA	fungal	neutral
Pleurotus euosmus	Steintaler Edelpilz	MEA	neutral	sweet
Pleurotus geesterani var.	Steintaler Edelpilz	MEA	fungal	sweet
Pleurotus nebrodensis var.	Steintaler Edelpilz	MEA	neutral	sweet
Pleurotus ostreatus I	DSMZ	MEA	fungal	sour
Pleurotus ostreatus II	Yoichi Honda	MEA	floral	neutral
Pleurotus ostreatus III	LCB	MEA	fungal	neutral
Pleurotus ostreatus IV	LCB	MEA	fungal	sweet
Pleurotus ostreatus var. V	Steintaler Edelpilz	MEA	fungal	sweet
Pleurotus ostreatus var.VI	Steintaler Edelpilz	MEA	fungal	sweet
Pleurotus ostreatus VII	Steintaler Edelpilz	MEA	fungal, floral	sweet, fungal
Pleurotus populinus	Steintaler Edelpilz	MEA	neutral	fermented
Pleurotus pulmonarius I	Steintaler Edelpilz	MEA	neutral	sweet
Pleurotus pulmonarius II	Göttingen	MEA	neutral	neutral
Pleurotus pulmonarius III	LCB	MEPA	fungal	floral, sweet
Pleurotus pulmonarius IV	CBS	MEPA	fruity, floral	floral
Pleurotus sajor-caju	DSMZ	MEA	fungal	fungal
Pleurotus salmoneo-stramineus	Göttingen	MEA	woodruff, fungal	woodruff
Pleurotus sapidus	DSMZ	MEA	woodruff	woodruff
Pleurotus spodoleucus	Steintaler Edelpilz	MEA	fungal, green	sweet, floral
Pleurotus tuberregium	Steintaler Edelpilz	MEA	fungal	sweet
Polyporus squamosus	CBS	MEA	fruity	sweet, citrus
Psathyrella candolleana	LCB	MEPA	neutral	sweet, fruity
Punctularia atropurpurascens	CBS	MEA	fungal	sweet
Punctularia strigosozonata	CBS	MEA	fungal	fungal
Pycnoporus cinnabarinus	DSMZ	MEPA	sour	fruity
Pycnoporus coccineus	CBS	MEA	sour	sweet
Pycnoporus sanguineus	DSMZ	MEA	fungal	citrus, sour
Sparassis crispa	LCB	MEA	sour, fungal	fungal
Strobilurus esculentus	LCB	MEPA	floral, fungal	fungal, green
Stropharia caerulea	DSMZ	MEPA	sweet, fruity	fungal
Stropharia rugosoannulata	Mycelia	MEA	floral	floral, sweet
Stropharia rugosoannulata	Mycelia	MEA	sweet	fruity, sweet
Suillus variegatus	DSMZ	MEA	chemical	sweet

Fungus	Source	Medium	OCA sensory	BCA sensory
Trametes ochracea	LCB	MEPA	fungal	fungal
Volvariella bombycina	Fraunhofer	MEA	floral, sour	neutral
Wolfiporia cocos	CBS	MEA	fungal	smokey

Table S2: Nutritional composition of the side streams of black carrot (BC) and orange carrot (OC) processing. Data received from SGS Institute Fresenius.

Value	BC	OC
Energy [kJ 100 g^{-1}]	1,007	1,035
Energy [kcal 100 g^{-1}]	237	244
Dry matter [g·100 g^{-1}]	70.46	69.34
Water [g·100 g^{-1}]	29.54	33.2
Fat [g·100 g^{-1}]	0.3	0.3
Protein [g·100 g^{-1}]	4.1	3.91
Carbohydrates [g·100 g^{-1}]	51.3	56.8
D-Fructose [g·100 g^{-1}]	13.8	30.66
D-Glucose [g·100 g^{-1}]	13.5	6.31
Sucrose [g·100 g^{-1}]	8.5	4.8
Fiber [g·100 g^{-1}]	3.61	2.28
Titratable acid (pH 8.1), calculated as citric acid [g·100 g^{-1}]	8.15	4.7
Ash [g·100 g^{-1}]	7.39	4.14
Sodium [mg·kg^{-1}]	7,300	3,630
Salt (calculated from sodium content) [g·100 g^{-1}]	1.83	0.908
Nitrate [mg·kg^{-1}]	630	2,900
Iron [mg·kg^{-1}]	20.9	22.6
Zinc [mg·kg^{-1}]	11.7	18.5
Manganese [mg·kg^{-1}]	9.67	13.5
Nitrite [mg·kg^{-1}]	5	5
Copper [mg·kg^{-1}]	1.13	3.02
Calculated sum of proteogenic amino acids [g·100 g^{-1}]	1.20	2.80
Proline [g·100 g^{-1}]	0.46	0.63
Alanine [g·100 g^{-1}]	0.2	0.53
γ-Aminobutyric acid [g·100 g^{-1}]	0.12	0.38
Arginine [g·100 g^{-1}]	0.09	0.38
Asparagine [g·100 g^{-1}]	0.08	0.37
Aspartic acid [g·100 g^{-1}]	0.07	0.27

Value	BC	OC
Valine [g·100 g^{-1}]	0.05	0.13
Glutamic acid [g·100 g^{-1}]	0.04	0.11
Isoleucine [g·100 g^{-1}]	0.03	0.07
Leucine [g·100 g^{-1}]	0.03	0.06
Phenylalanine [g·100 g^{-1}]	0.02	0.05
Serine [g·100 g^{-1}]	0.02	0.03
Threonine [g·100 g^{-1}]	0.02	0.03
Tyrosine [g·100 g^{-1}]	0.02	0.03
Cysteine [g·100 g^{-1}]	0.01	0.02
Glutamine [g·100 g^{-1}]	0.01	0.02
Glycine [g·100 g^{-1}]	0.01	0.02
Histidine [g·100 g^{-1}]	0.01	0.02
Hydroxylysine [g·100 g^{-1}]	0.01	0.01
Hydroxyproline [g·100 g^{-1}]	0.01	0.01
Lysine [g·100 g^{-1}]	0.01	0.01
Methionine [g·100 g^{-1}]	0.01	0.01
Ornithine [g·100 g^{-1}]	0.01	0.01
Taurine [g·100 g^{-1}]	0.01	0.01
Tryptophane [g·100 g^{-1}]	0.01	0.01

Table S3: Fungi screened in submerged cultures in 2.2% carbohydrate orange carrot medium (OCM) and 1.8% carbohydrate black carrot medium (BCM) and the day with their highest dry matter (DM).

Fungi	Highest DM in OCM	Highest DM in BCM
Agaricus arvensis	10	-
Caloycbe gambosa	5	3
Fistulina hepatica	10	-
Laetiporus persicinus	4	7
Laetiporus sulphureus	9	-
Lentinula edodes	7	4
Meripilus giganteus II	7	9
Mycetinis scorodonius	6	6
Pleurotus citrinopileatus	9	-
Pleurotus djamor var.	6	10
Pleurotus eryngii	10	10
Pleurotus geesterani var.	3	10
Pleurotus ostreatus I	9	10
Pleurotus ostreatus II	-	10
Pleurotus ostreatus IV	6	10
Pleurotus ostreatus var. V	8	10

Pleurotus ostreatus var. VI	5	7
Pleurotus sajor-caju	8	-
Pleurotus salmoneo-stramineus	7	8
Pleurotus sapidus	3	9
Pleurotus spodoleucus	5	-
Pleurotus pulmonarius	4	10
Wolfiporia cocos	3	7

Table S4: Example of the Design of Experiment for the optimization of the submerged cultures in black carrot medium (BCM) and orange carrot medium (OCM). CH: carbohydrate DM: dry matter CP: crude protein

Run	**Factor 1**		**Factor 2**	**Result 1**	**Result 2**
	**B: CH content**		**C: pH**	***DM***	***CP***
	**%**		**-**	**g 100 mL^{-1}**	**g 100 g^{-1}**
	**OCM**	**BCM**			
1	1.7	1.3	7.9		
2	3.2	2.6	7.5		
3	2.1	1.7	5.6		
4	5.6	4.5	7.9		
5	1.7	1.3	7.9		
6	3.6	2.9	6.0		
7	1.7	1.3	7.9		
8	5.2	4.1	6.4		
9	3.6	2.9	9.0		
10	5.6	4.5	4.1		
11	3.6	2.9	6.0		
12	4.1	3.3	4.5		
13	3.6	2.9	3.0		
14	3.2	2.6	7.5		
15	1.7	1.3	4.1		
16	4.1	3.3	4.5		
17	3.6	2.9	6.0		
18	0.6	0.4	6.0		
19	5.6	4.5	7.9		
20	0.6	0.4	6.0		
21	6.7	5.4	6.0		
22	3.6	2.9	6.0		
23	2.1	1.7	5.6		
24	3.6	2.9	3.0		
25	5.2	4.1	6.4		
26	3.6	2.9	9.0		
27	1.7	1.3	4.1		
28	5.6	4.5	4.1		
29	5.6	4.5	7.9		

30	6.7	5.4	6.0		
31	5.6	4.5	4.1		
32	1.7	1.3	4.1		
33	3.6	2.9	6.0		
34	3.6	2.9	6.0		

Table S5: Statistic values of the Design of Experiment for dry matter of *Agaricus arvensis* in orange carrot media.

Source	Sum of Squares	df	Mean Square	F-value	p-value	
**Model**	1.96	7	0.2799	56.55	< 0.0001	significant
A-Source medium	0.1939	1	0.1939	39.18	< 0.0001	
B-pH-value	0.1881	1	0.1881	38.00	< 0.0001	
AB	0.0057	1	0.0057	1.16	0.2957	
A^2	0.5291	1	0.5291	106.90	< 0.0001	
B^2	0.2904	1	0.2904	58.67	< 0.0001	
AB^2	0.2097	1	0.2097	42.37	< 0.0001	
B^3	0.1377	1	0.1377	27.82	< 0.0001	
**Residual**	0.0940	19	0.0049			
Lack of Fit	0.0380	4	0.0095	2.54	0.0833	not significant
Pure Error	0.0561	15	0.0037			
**Cor Total**	2.05	26				
**Std. Dev.**	0.0704		**R^2**	0.9542		
**Mean**	0.6015		**Adjusted R^2**	0.9373		
**C.V. %**	11.70		**Predicted R^2**	0.9152		
			**Adeq Precision**	20.3018		

Table S6: Statistic values of the Design of Experiment for crude protein of *Agaricus arvensis* in orange carrot media.

Source	Sum of Squares	df	Mean Square	F-value	p-value	
**Model**	86.77	8	10.85	177.23	< 0.0001	significant
A- Source medium	0.0702	1	0.0702	1.15	0.2983	
B-pH-value	0.4226	1	0.4226	6.91	0.0171	
AB	0.0965	1	0.0965	1.58	0.2252	
A^2	12.72	1	12.72	207.87	< 0.0001	
B^2	11.98	1	11.98	195.76	< 0.0001	
AB^2	6.29	1	6.29	102.74	< 0.0001	
A^3	2.33	1	2.33	38.03	< 0.0001	
B^3	0.9764	1	0.9764	15.96	0.0009	
**Residual**	1.10	18	0.0612			
Lack of Fit	0.8711	3	0.2904	18.90	< 0.0001	significant
Pure Error	0.2305	15	0.0154			
**Cor Total**	87.88	26				
**Std. Dev.**	0.2474		**R^2**	0.9875		
**Mean**	2.68		**Adjusted R^2**	0.9819		
**C.V. %**	9.24		**Predicted R^2**	0.9559		
			**Adeq Precision**	30.4300		

Table S7: Statistic values of the Design of Experiment for dry matter of *Pleurotus djamor* in orange carrot media.

Source	Sum of Squares	df	Mean Square	F-value	p-value	
**Model**	23.75	7	3.39	14.44	< 0.0001	significant
A-Soruce medium	5.67	1	5.67	24.13	< 0.0001	
B-pH-value	4.23	1	4.23	18.00	0.0003	
AB	1.23	1	1.23	5.24	0.0312	
A^2	0.6583	1	0.6583	2.80	0.1071	
B^2	1.73	1	1.73	7.36	0.0121	
A^3	0.8622	1	0.8622	3.67	0.0674	
B^3	5.52	1	5.52	23.49	< 0.0001	
**Residual**	5.64	24	0.2349			
Lack of Fit	4.22	5	0.8448	11.35	< 0.0001	significant
Pure Error	1.41	19	0.0744			
**Cor Total**	29.39	31				
**Std. Dev.**	0.4847		**R^2**	0.8082		
**Mean**	1.86		**Adjusted R^2**	0.7522		
**C.V. %**	26.04		**Predicted R^2**	0.5899		
			**Adeq Precision**	11.5913		

Table S8: Statistic values of the Design of Experiment for crude protein of *Pleurotus djamor* in orange carrot media.

Source	Sum of Squares	df	Mean Square	F-value	p-value	
**Model**	295.16	7	42.17	72.97	< 0.0001	significant
A-Source medium	0.0034	1	0.0034	0.0060	0.9391	
B-pH-value	0.0197	1	0.0197	0.0341	0.8549	
AB	3.97	1	3.97	6.86	0.0150	
A^2	6.08	1	6.08	10.51	0.0035	
B^2	70.16	1	70.16	121.42	< 0.0001	
A^3	6.92	1	6.92	11.98	0.0020	
B^3	30.15	1	30.15	52.17	< 0.0001	
**Residual**	13.87	24	0.5778			
Lack of Fit	6.15	5	1.23	3.02	0.0357	significant
Pure Error	7.72	19	0.4065			
**Cor Total**	309.02	31				
**Std. Dev.**	0.7602		**R^2**	0.9551		
**Mean**	10.08		**Adjusted R^2**	0.9420		
**C.V. %**	7.54		**Predicted R^2**	0.9027		
			**Adeq Precision**	39.1571		

Table S9: Statistic values of the Design of Experiment for dry matter of *Pleurotus geesterani* in orange carrot media.

Source	Sum of Squares	df	Mean Square	F-value	p-value	
**Model**	2.30	7	0.3283	10.93	< 0.0001	significant
A-Source medium	0.0033	1	0.0033	0.1103	0.7425	
B-pH-value	0.2667	1	0.2667	8.88	0.0062	
AB	0.0295	1	0.0295	0.9840	0.3304	
A^2	0.3344	1	0.3344	11.14	0.0026	
B^2	1.09	1	1.09	36.32	< 0.0001	
A^2B	0.3297	1	0.3297	10.98	0.0027	
A^3	0.2056	1	0.2056	6.85	0.0146	
**Residual**	0.7807	26	0.0300			
Lack of Fit	0.1484	5	0.0297	0.9855	0.4498	not significant
Pure Error	0.6323	21	0.0301			
**Cor Total**	3.08	33				
**Std. Dev.**	0.1733		**R^2**	0.7464		
**Mean**	0.6818		**Adjusted R^2**	0.6782		
**C.V. %**	25.42		**Predicted R^2**	0.5758		
			**Adeq Precision**	12.2723		

Table S10: Statistic values of the Design of Experiment for crude protein of *Pleurotus geesterani* in orange carrot media.

Source	Sum of Squares	df	Mean Square	F-value	p-value	
**Model**	586.24	4	146.56	9.29	< 0.0001	significant
A-Source medium	7.80	1	7.80	0.4945	0.4875	
B-pH-value	145.84	1	145.84	9.24	0.0050	
A^2	73.89	1	73.89	4.68	0.0388	
B^2	376.63	1	376.63	23.87	< 0.0001	
**Residual**	457.49	29	15.78			
Lack of Fit	339.65	8	42.46	7.57	< 0.0001	significant
Pure Error	117.84	21	5.61			
**Cor Total**	1043.72	33				
**Std. Dev.**	3.97		**R^2**	0.5617		
**Mean**	17.12		**Adjusted R^2**	0.5012		
**C.V. %**	23.20		**Predicted R^2**	0.3834		
			**Adeq Precision**	9.8051		

Table S11: Statistic values of the Design of Experiment for dry matter of *Pleurotus ostreatus* var. VI in orange carrot media.

Source	Sum of Squares	df	Mean Square	F-value	p-value	
**Model**	6.47	8	0.8087	11.68	< 0.0001	significant
A-Source medium	1.22	1	1.22	17.67	0.0003	
B-pH-value	0.3904	1	0.3904	5.64	0.0256	
AB	0.0458	1	0.0458	0.6608	0.4239	
A^2	0.2170	1	0.2170	3.13	0.0889	
B^2	1.38	1	1.38	19.97	0.0001	
AB^2	0.5213	1	0.5213	7.53	0.0111	
A^3	0.2706	1	0.2706	3.91	0.0592	
B^3	0.5450	1	0.5450	7.87	0.0096	
**Residual**	1.73	25	0.0693			
Lack of Fit	0.9183	4	0.2296	5.93	0.0023	significant
Pure Error	0.8130	21	0.0387			
**Cor Total**	8.20	33				
**Std. Dev.**	0.2632		**R^2**	0.7889		
**Mean**	1.04		**Adjusted R^2**	0.7213		
**C.V. %**	25.25		**Predicted R^2**	0.5971		
			**Adeq Precision**	11.6605		

Table S12: Statistic values of the Design of Experiment for crude protein of *Pleurotus ostreatus* var. VI in orange carrot media.

**Source**	**Sum of Squares**	**df**	**Mean Square**	**F-value**	**p-value**	
**Model**	521.03	7	74.43	87.92	< 0.0001	significant
A-Source medium	67.93	1	67.93	80.24	< 0.0001	
B-pH-value	0.5285	1	0.5285	0.6242	0.4372	
AB	1.06	1	1.06	1.25	0.2742	
A^2	53.96	1	53.96	63.73	< 0.0001	
B^2	59.76	1	59.76	70.59	< 0.0001	
AB^2	8.17	1	8.17	9.65	0.0048	
B^3	55.76	1	55.76	65.86	< 0.0001	
**Residual**	20.32	24	0.8466			
Lack of Fit	4.63	5	0.9260	1.12	0.3824	not significant
Pure Error	15.69	19	0.8257			
**Cor Total**	541.35	31				
**Std. Dev.**	0.9201		**R^2**	0.9625		
**Mean**	12.69		**Adjusted R^2**	0.9515		
**C.V. %**	7.25		**Predicted R^2**	0.9384		
			**Adeq Precision**	42.1985		

Table S13: Statistic values of the Design of Experiment for dry matter of *Pleurotus spodoleucus* in orange carrot media.

Source	Sum of Squares	df	Mean Square	F-value	p-value	
**Model**	16.77	8	2.10	80.26	< 0.0001	significant
A-Source medium	1.69	1	1.69	64.78	< 0.0001	
B-pH-value	0.0026	1	0.0026	0.0986	0.7562	
AB	0.5097	1	0.5097	19.51	0.0002	
A^2	0.0006	1	0.0006	0.0218	0.8838	
B^2	1.27	1	1.27	48.46	< 0.0001	
AB^2	0.2416	1	0.2416	9.25	0.0056	
A^3	0.1305	1	0.1305	5.00	0.0350	
B^3	0.3939	1	0.3939	15.08	0.0007	
**Residual**	0.6269	24	0.0261			
Lack of Fit	0.1974	4	0.0494	2.30	0.0945	not significant
Pure Error	0.4295	20	0.0215			
**Cor Total**	17.40	32				
**Std. Dev.**	0.1616		**R^2**	0.9640		
**Mean**	1.42		**Adjusted R^2**	0.9520		
**C.V. %**	11.40		**Predicted R^2**	0.9270		
			**Adeq Precision**	30.1714		

Table S14: Statistic values of the Design of Experiment for crude protein of *Pleurotus spodoleucus* in orange carrot media.

Source	Sum of Squares	df	Mean Square	F-value	p-value	
**Model**	306.13	5	61.23	18.19	< 0.0001	significant
A-Source medium	23.56	1	23.56	7.00	0.0132	
B-pH-value	22.54	1	22.54	6.70	0.0151	
AB	17.62	1	17.62	5.24	0.0299	
B^2	93.10	1	93.10	27.66	< 0.0001	
B^3	103.08	1	103.08	30.63	< 0.0001	
**Residual**	94.24	28	3.37			
Lack of Fit	71.58	7	10.23	9.48	< 0.0001	significant
Pure Error	22.66	21	1.08			
**Cor Total**	400.37	33				
**Std. Dev.**	1.83		**R^2**	0.7646		
**Mean**	12.15		**Adjusted R^2**	0.7226		
**C.V. %**	15.10		**Predicted R^2**	0.5867		
			**Adeq Precision**	17.4248		

Table S15: Statistic values of the Design of Experiment for dry matter of *Pleurotus djamor* in black carrot media**.**

Source	Sum of Squares	df	Mean Square	F-value	p-value	
Block	3.34	1	3.34			
**Model**	7.98	9	0.8866	68.56	< 0.0001	significant
A-Source medium	0.4899	1	0.4899	37.89	< 0.0001	
B-pH-value	0.0059	1	0.0059	0.4592	0.5027	
AB	1.32	1	1.32	102.34	< 0.0001	
A^2	2.19	1	2.19	169.66	< 0.0001	
B^2	2.11	1	2.11	163.26	< 0.0001	
A^2B	0.2046	1	0.2046	15.83	0.0004	
AB^2	0.9522	1	0.9522	73.64	< 0.0001	
A^3	0.3844	1	0.3844	29.73	< 0.0001	
B^3	0.6315	1	0.6315	48.83	< 0.0001	
**Residual**	0.4267	33	0.0129			
Lack of Fit	0.2293	6	0.0382	5.23	0.0011	significant
Pure Error	0.1974	27	0.0073			
**Cor Total**	11.74	43				
**Std. Dev.**	0.1137		**R^2**	0.9492		
**Mean**	0.8995		**Adjusted R^2**	0.9354		
**C.V. %**	12.64		**Predicted R^2**	0.9076		
			**Adeq Precision**	26.1922		

Table S16: Statistic values of the Design of Experiment for crude protein of *Pleurotus djamor* in black carrot media.

Source	Sum of Squares	df	Mean Square	F-value	p-value	
Block	43.00	1	43.00			
**Model**	64.03	9	7.11	69.62	< 0.0001	significant
A-Source medium	2.94	1	2.94	28.81	< 0.0001	
B-pH-value	0.0047	1	0.0047	0.0459	0.8317	
AB	9.54	1	9.54	93.38	< 0.0001	
A^2	3.44	1	3.44	33.70	< 0.0001	
B^2	15.71	1	15.71	153.74	< 0.0001	
A^2B	0.9390	1	0.9390	9.19	0.0047	
AB^2	9.89	1	9.89	96.76	< 0.0001	
A^3	5.10	1	5.10	49.89	< 0.0001	
B^3	6.34	1	6.34	62.04	< 0.0001	
**Residual**	3.37	33	0.1022			
Lack of Fit	3.19	6	0.5319	79.46	< 0.0001	significant
Pure Error	0.1808	27	0.0067			
**Cor Total**	110.40	43				
**Std. Dev.**	0.3197		**R^2**	0.9500		
**Mean**	2.32		**Adjusted R^2**	0.9363		
**C.V. %**	13.79		**Predicted R^2**	0.9133		
			**Adeq Precision**	25.8582		

Table S17: Statistic values of the Design of Experiment for dry matter of *Meripilus giganteus* II in black carrot media.

Source	Sum of Squares	df	Mean Square	F-value	p-value	
**Model**	55.10	8	6.89	28.66	< 0.0001	significant
A-Source medium	5.71	1	5.71	23.76	< 0.0001	
B-pH-value	16.81	1	16.81	69.94	< 0.0001	
AB	2.57	1	2.57	10.69	0.0031	
A^2	0.7311	1	0.7311	3.04	0.0934	
B^2	10.00	1	10.00	41.62	< 0.0001	
A^2B	7.78	1	7.78	32.37	< 0.0001	
AB^2	3.40	1	3.40	14.14	0.0009	
B^3	11.87	1	11.87	49.41	< 0.0001	
**Residual**	6.01	25	0.2403			
Lack of Fit	2.05	4	0.5134	2.73	0.0568	not significant
Pure Error	3.95	21	0.1883			
**Cor Total**	61.11	33				
**Std. Dev.**	0.4902		**R^2**	0.9017		
**Mean**	-2.18		**Adjusted R^2**	0.8702		
**C.V. %**	22.48		**Predicted R^2**	0.8298		
			**Adeq Precision**	16.9574		

Table S18: Statistic values of the Design of Experiment for crude protein of *Meripilus giganteus* Ilin black carrot media.

Source	Sum of Squares	df	Mean Square	F-value	p-value	
**Model**	1584.77	8	198.10	59.05	< 0.0001	significant
A-Source medium	419.05	1	419.05	124.92	< 0.0001	
B-pH-value	350.83	1	350.83	104.58	< 0.0001	
AB	135.39	1	135.39	40.36	< 0.0001	
A^2	16.58	1	16.58	4.94	0.0386	
B^2	104.95	1	104.95	31.29	< 0.0001	
A^2B	279.44	1	279.44	83.30	< 0.0001	
A^3	44.68	1	44.68	13.32	0.0017	
B^3	287.88	1	287.88	85.82	< 0.0001	
**Residual**	63.74	19	3.35			
Lack of Fit	59.56	3	19.85	76.05	< 0.0001	significant
Pure Error	4.18	16	0.2610			
**Cor Total**	1648.50	27				
**Std. Dev.**	1.83		**R^2**	0.9613		
**Mean**	6.62		**Adjusted R^2**	0.9451		
**C.V. %**	27.65		**Predicted R^2**	0.9097		
			**Adeq Precision**	18.2046		

Table S19: Statistic values of the Design of Experiment for dry matter of *Pleurotus ostreatus* var. V in black carrot media.

Source	Sum of Squares	df	Mean Square	F-value	p-value	
**Model**	81.22	8	10.15	31.53	< 0.0001	significant
A-Source medium	0.6330	1	0.6330	1.97	0.1737	
B-pH-value	5.16	1	5.16	16.01	0.0005	
AB	0.0139	1	0.0139	0.0430	0.8374	
A^2	27.26	1	27.26	84.66	< 0.0001	
B^2	33.92	1	33.92	105.37	< 0.0001	
AB^2	12.11	1	12.11	37.61	< 0.0001	
A^3	4.66	1	4.66	14.48	0.0009	
B^3	7.91	1	7.91	24.56	< 0.0001	
**Residual**	7.73	24	0.3219			
Lack of Fit	5.40	4	1.35	11.57	< 0.0001	significant
Pure Error	2.33	20	0.1165			
**Cor Total**	88.95	32				
**Std. Dev.**	0.5674		**R^2**	0.9131		
**Mean**	-0.9511		**Adjusted R^2**	0.8842		
**C.V. %**	59.66		**Predicted R^2**	0.8361		
			**Adeq Precision**	14.613 8		

Table S20: Statistic values of the Design of Experiment for crude protein of *Pleurotus ostreatus* var. V in black carrot media.

**Source**	**Sum of Squares**	**df**	**Mean Square**	**F-value**	**p-value**	
**Model**	62.63	9	6.96	202.83	< 0.0001	significant
A-Source medium	0.5372	1	0.5372	15.66	0.0007	
B-pH-value	0.0031	1	0.0031	0.0900	0.7672	
AB	0.6588	1	0.6588	19.20	0.0003	
A^2	5.47	1	5.47	159.54	< 0.0001	
B^2	4.04	1	4.04	117.67	< 0.0001	
A^2B	0.2143	1	0.2143	6.25	0.0208	
AB^2	0.2544	1	0.2544	7.42	0.0127	
A^3	0.8336	1	0.8336	24.30	< 0.0001	
B^3	1.85	1	1.85	54.05	< 0.0001	
**Residual**	0.7205	21	0.0343			
Lack of Fit	0.3234	2	0.1617	7.74	0.0035	significant
Pure Error	0.3971	19	0.0209			
**Cor Total**	63.35	30				
**Std. Dev.**	0.1852		**R^2**	0.9886		
**Mean**	2.70		**Adjusted R^2**	0.9838		
**C.V. %**	6.86		**Predicted R^2**	0.9725		
			**Adeq Precision**	37.5478		

Table S21: Statistic values of the Design of Experiment for dry matter of *Pleurotus ostreatus* var. VI in black carrot media.

Source	Sum of Squares	df	Mean Square	F-value	p-value	
**Model**	48.82	7	6.97	29.67	< 0.0001	significant
A-Source medium	0.8942	1	0.8942	3.80	0.0624	
B-pH-value	0.2252	1	0.2252	0.9579	0.3371	
AB	0.0003	1	0.0003	0.0014	0.9707	
A^2	13.37	1	13.37	56.86	< 0.0001	
B^2	33.76	1	33.76	143.64	< 0.0001	
AB^2	9.70	1	9.70	41.25	< 0.0001	
A^3	2.43	1	2.43	10.33	0.0036	
**Residual**	5.88	25	0.2351			
Lack of Fit	4.59	5	0.9173	14.22	< 0.0001	significant
Pure Error	1.29	20	0.0645			
**Cor Total**	54.70	32				
**Std. Dev.**	0.4848		**R^2**	0.8926		
**Mean**	-1.40		**Adjusted R^2**	0.8625		
**C.V. %**	34.61		**Predicted R^2**	0.8371		
			**Adeq Precision**	14.4209		

Table S22: Statistic values of the Design of Experiment for crude protein of *Pleurotus ostreatus* var. VI in black carrot media.

Source	Sum of Squares	df	Mean Square	F-value	p-value	
**Model**	94.94	7	13.56	118.78	< 0.0001	significant
A-Source medium	0.8792	1	0.8792	7.70	0.0103	
B-pH-value	0.0545	1	0.0545	0.4773	0.4960	
AB	0.4847	1	0.4847	4.25	0.0499	
A^2	5.95	1	5.95	52.08	< 0.0001	
B^2	37.72	1	37.72	330.33	< 0.0001	
AB^2	3.05	1	3.05	26.68	< 0.0001	
A^3	0.8469	1	0.8469	7.42	0.0116	
**Residual**	2.85	25	0.1142			
Lack of Fit	1.95	5	0.3895	8.59	0.0002	significant
Pure Error	0.9068	20	0.0453			
**Cor Total**	97.79	32				
**Std. Dev.**	0.3379		**R^2**	0.9708		
**Mean**	2.51		**Adjusted R^2**	0.9626		
**C.V. %**	13.45		**Predicted R^2**	0.9529		
			**Adeq Precision**	27.8814		

Table S23: Statistic values of the Design of Experiment for dry matter of *Pleurotus geesterani* in black carrot media.

Source	Sum of Squares	df	Mean Square	F-value	p-value	
**Model**	3.12	6	0.5197	35.39	< 0.0001	significant
A-Source medium	0.0657	1	0.0657	4.47	0.0438	
B-pH-value	0.0042	1	0.0042	0.2854	0.5976	
AB	0.0011	1	0.0011	0.0774	0.7830	
A^2	1.28	1	1.28	87.22	< 0.0001	
B^2	2.24	1	2.24	152.69	< 0.0001	
AB^2	0.4053	1	0.4053	27.60	< 0.0001	
**Residual**	0.3965	27	0.0147			
Lack of Fit	0.2558	6	0.0426	6.36	0.0006	significant
Pure Error	0.1407	21	0.0067			
**Cor Total**	3.51	33				
**Std. Dev.**	0.1212		**R^2**	0.8872		
**Mean**	0.6626		**Adjusted R^2**	0.8621		
**C.V. %**	18.29		**Predicted R^2**	0.8208		
			**Adeq Precision**	14.6543		

Table S24: Statistic values of the Design of Experiment for crude protein of *Pleurotus geesterani* in black carrot media.

Source	Sum of Squares	df	Mean Square	F-value	p-value	
**Model**	132.70	7	18.96	123.56	< 0.0001	significant
A-Source medium	0.2349	1	0.2349	1.53	0.2270	
B-pH-value	0.0064	1	0.0064	0.0416	0.8399	
AB	0.2110	1	0.2110	1.38	0.2515	
A^2	13.75	1	13.75	89.60	< 0.0001	
B^2	60.25	1	60.25	392.67	< 0.0001	
AB^2	6.61	1	6.61	43.08	< 0.0001	
A^3	2.45	1	2.45	15.97	0.0005	
**Residual**	3.99	26	0.1534			
Lack of Fit	3.82	5	0.7639	94.59	< 0.0001	significant
Pure Error	0.1696	21	0.0081			
**Cor Total**	136.69	33				
**Std. Dev.**	0.3917		**R^2**	0.9708		
**Mean**	2.85		**Adjusted R^2**	0.9630		
**C.V. %**	13.74		**Predicted R^2**	0.9568		
			**Adeq Precision**	25.7801		

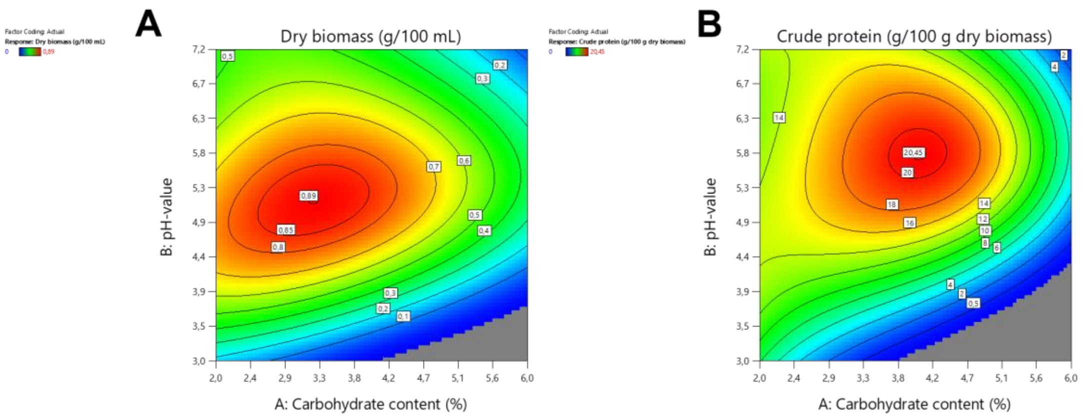

Figure S1: Response surface plot for *Agaricus arvensis* in orange carrot media for dry biomass (A) and crude protein content (B)

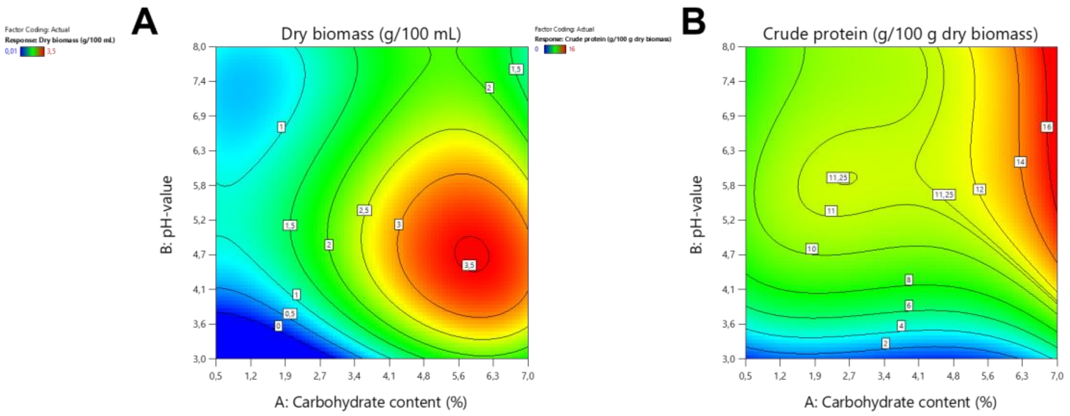

Figure S2: Response surface plot for *Pleurotus djamor* in orange carrot media for dry biomass (A) and crude protein content (B).

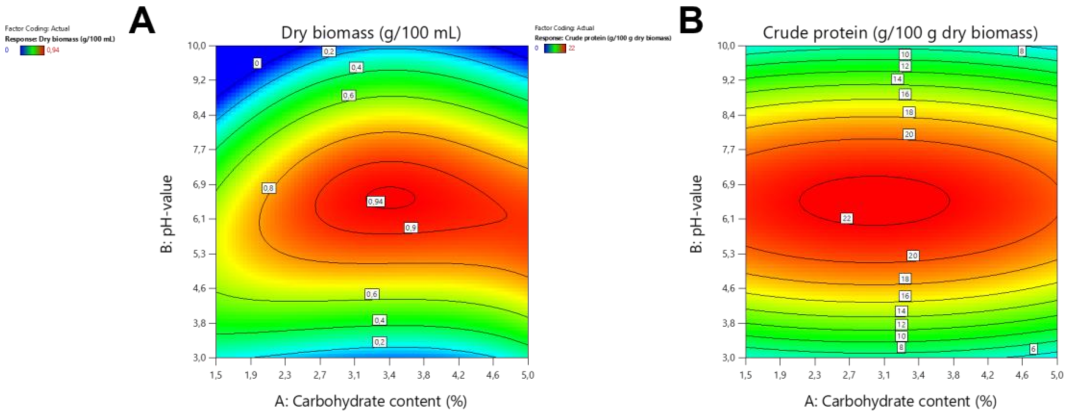

Figure S3: Response surface plot for *Pleurotus geesterani* in orange carrot media for dry biomass (A) and crude protein content (B).

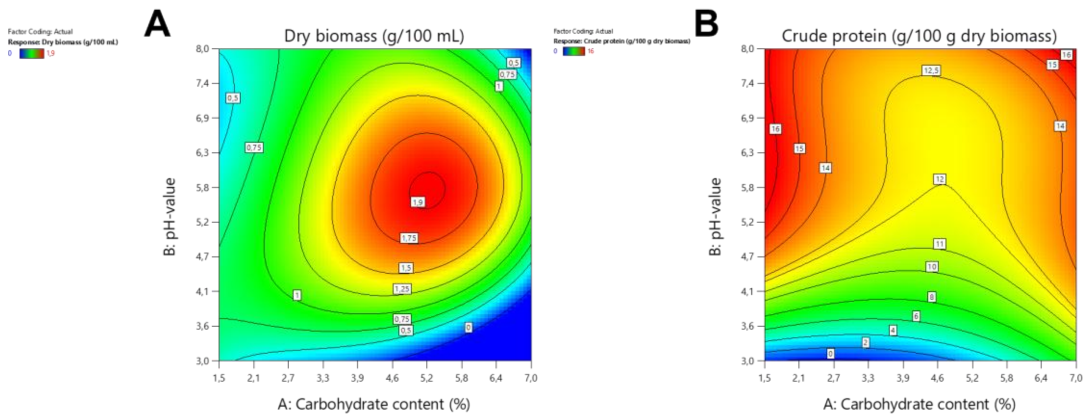

Figure S4: Response surface plot for *Pleurotus ostreatus* var. VI in orange carrot media for dry biomass (A) and crude protein content (B).

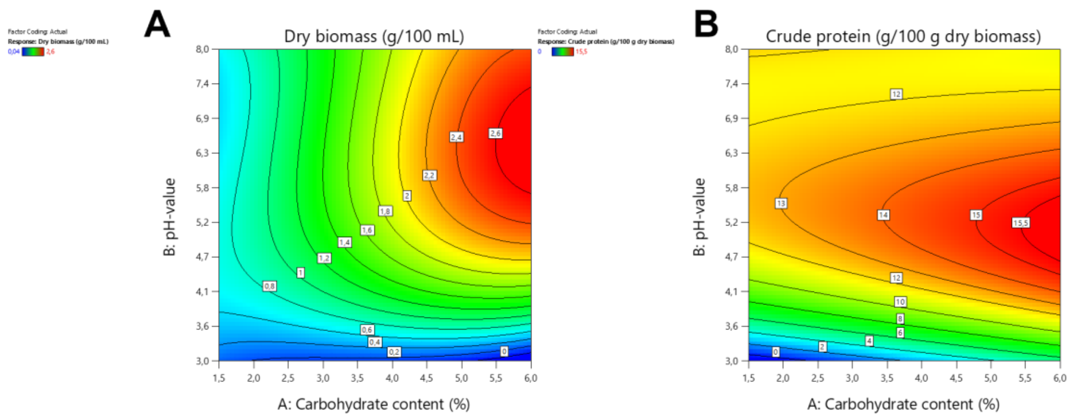

Figure S5: Response surface plot for *Pleurotus spodoleucus* in orange carrot media for dry biomass (A) and crude protein content (B).

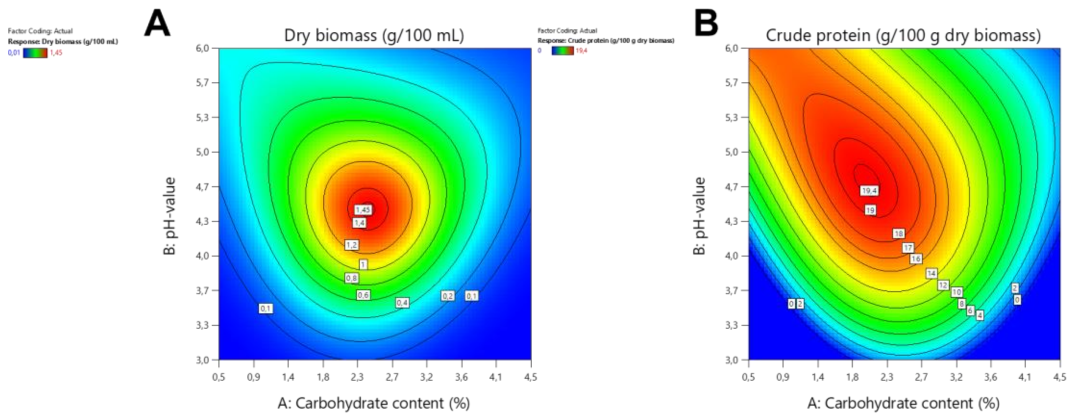

Figure S6: Response surface plot for *Meripilus giganteus* II in black carrot media for dry biomass (A) and crude protein content (B).

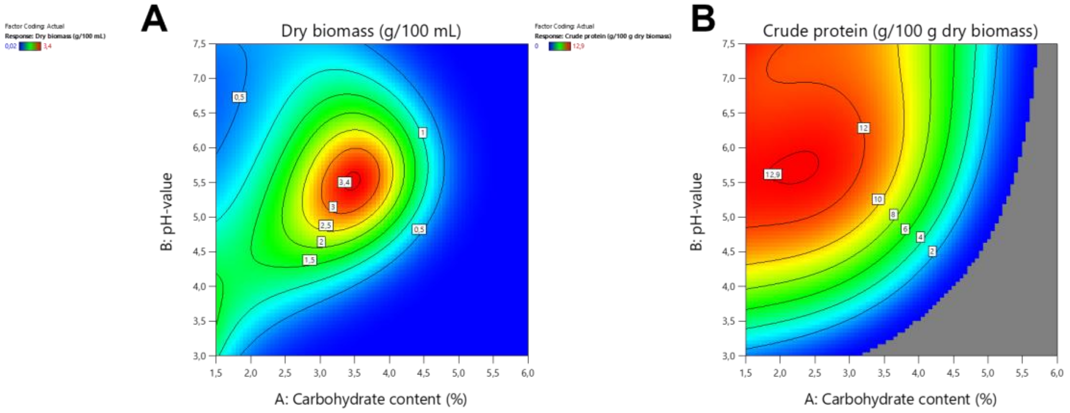

Figure S7: Response surface plot for *Pleurotus ostreatus* var. V in black carrot media for dry biomass (A) and crude protein content (B).

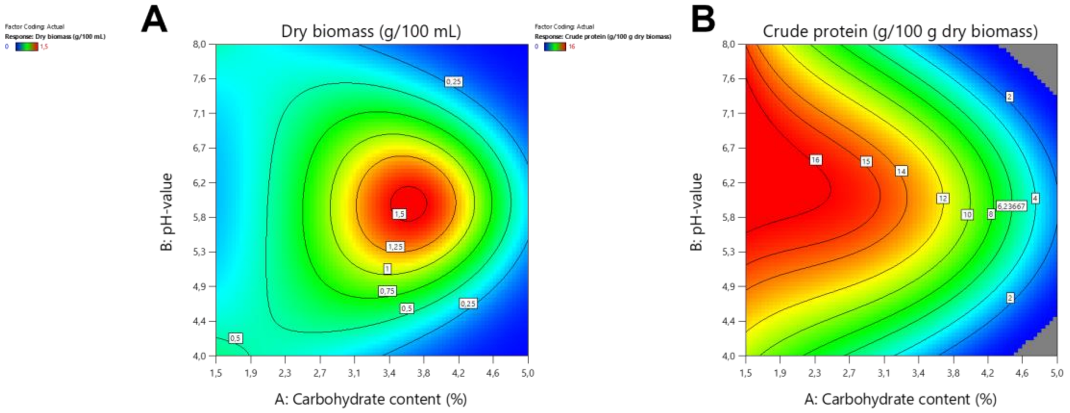

Figure S8: Response surface plot for *Pleurotus ostreatus* var. VI in black carrot media for dry biomass (A) and crude protein content (B).

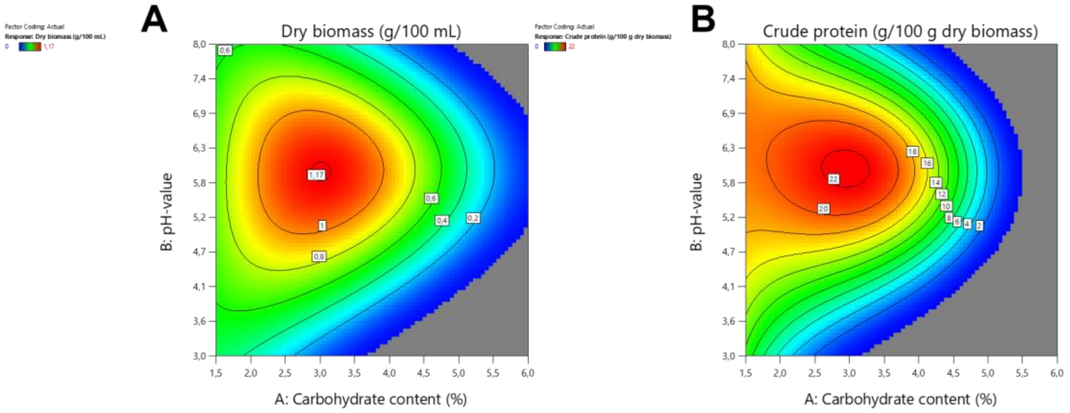

Figure S9: Response surface plot for *Pleurotus geesterani* in black carrot media for dry biomass (A) and crude protein content (B).

References

(1) Matissek, R.; Fischer, M. *Lebensmittelanalytik*, 7th ed.; Springer Berlin Heidelberg; Imprint: Springer Spektrum: Berlin, Heidelberg, 2021.
